# Altered DNA methylome profiles of blood leukocytes in Chinese patients with mild cognitive impairment and Alzheimer’s disease

**DOI:** 10.3389/fgene.2023.1175864

**Published:** 2023-06-14

**Authors:** Shaochang Wu, Fan Yang, Shan Chao, Bo Wang, Wuqian Wang, He Li, Limei Yu, Lin He, Xingwang Li, Liya Sun, Shengying Qin

**Affiliations:** ^1^Department of Geriatrics, Lishui Second People’s Hospital, Lishui, China; ^2^Key Laboratory of Cell Engineering in Guizhou Province, Affiliated Hospital of Zunyi Medical University, Zunyi, China; ^3^ Bio-X Institutes, Key Laboratory for the Genetics of Developmental and Neuropsychiatric Disorders, Ministry of Education, Shanghai Jiao Tong University, Shanghai, China; ^4^ Research Center for Lin He Academician New Medicine, Institutes for Shanghai Pudong Decoding Life, Shanghai, China; ^5^Department of Obstetrics and Gynecology, Key Laboratory for Major Obstetric Diseases of Guangdong Province, The Third Affiliated Hospital of Guangzhou Medical University, Guangzhou, China; ^6^ Shanghai Mental Health Center, Editorial Office, School of Medicine, Shanghai Jiao Tong University, Shanghai, China

**Keywords:** mild cognitive impairment, Alzheimer’s disease, blood DNA methylome profile, differentially methylated CpG sites, potential biomarker

## Abstract

**Objective:** DNA methylation plays a potential role in the pathogenesis of Alzheimer’s disease (AD). However, little is known about the global changes of blood leukocyte DNA methylome profiles from Chinese patients with mild cognitive impairment (MCI) and with AD, or the specific DNA methylation-based signatures associated with MCI and AD. In this study, we sought to dissect the characteristics of blood DNA methylome profiles in MCI- and AD-affected Chinese patients with the aim of identifying novel DNA methylation biomarkers for AD.

**Methods:** In this study, we profiled the DNA methylome of peripheral blood leukocytes from 20 MCI- and 20 AD-affected Chinese patients and 20 cognitively healthy controls (CHCs) with the Infinium Methylation EPIC BeadChip array.

**Results:** We identified significant alterations of the methylome profiles in MCI and AD blood leukocytes. A total of 2,582 and 20,829 CpG sites were significantly and differentially methylated in AD and MCI compared with CHCs (adjusted *p* < 0.05), respectively. Furthermore, 441 differentially methylated positions (DMPs), aligning to 213 unique genes, were overlapped by the three comparative groups of AD *versus* CHCs, MCI *versus* CHCs, and AD *versus* MCI, of which 6 and 5 DMPs were continuously hypermethylated and hypomethylated in MCI and AD relative to CHCs (adjusted *p* < 0.05), respectively, such as *FLNC* cg20186636 and *AFAP1* cg06758191. The DMPs with an area under the curve >0.900, such as cg18771300, showed high potency for predicting MCI and AD. In addition, gene ontology and pathway enrichment results showed that these overlapping genes were mainly involved in neurotransmitter transport, GABAergic synaptic transmission, signal release from synapse, neurotransmitter secretion, and the regulation of neurotransmitter levels. Furthermore, tissue expression enrichment analysis revealed a subset of potentially cerebral cortex-enriched genes associated with MCI and AD, including *SYT7*, *SYN3*, and *KCNT1.*

**Conclusion:** This study revealed a number of potential biomarkers for MCI and AD, also highlighted the presence of epigenetically dysregulated gene networks that may engage in the underlying pathological events resulting in the onset of cognitive impairment and AD progression. Collectively, this study provides prospective cues for developing therapeutic strategies to improve cognitive impairment and AD course.

## 1 Introduction

Dementia is a common syndrome characterized by deterioration in cognitive function, in which memory, language, thinking, comprehension, as well as judgement and learning capacity are often affected. According to the latest data of dementia epidemiology released by World Health Organization (https://www.who.int/news-room/fact-sheets/detail/dementia). Currently there are approximately 55 million people affected by dementia worldwide, especially in low- and middle-income countries. Moreover, almost 10 million new cases are diagnosed every year, this number is estimated to rise to 66 million and 131 million by 2030 and 2050 (Livingston et al., 2017), respectively. As a public health priority, dementia has prompted the World Health Assembly to endorse the *Global Action Plan on The Public Health Response to Dementia 2017–2025*, in May 2017. In China, approximately 15.07 million individuals aged 60 years and older are affected by dementia currently, of which 9.83 million are AD cases, 3.92 million are vascular dementia cases and 1.32 million are other types of dementia (Ren et al., 2022). Furthermore, approximately 38.77 million individuals are affected by mild cognitive impairment (MCI) among Chinese populations over 60 years of age (Jia et al., 2020a). In response, the Chinese government has launched a battery of plans including ‘Healthy China Action’ Plan of 2019–2030 and related policies of the 13th Five-Year Plan, to better manage dementia and related disorders (Jia et al., 2020b; Ren et al., 2022). There are many different forms of dementia, such as Alzheimer’s disease (AD), dementia with Lewy bodies, frontotemporal dementia and vascular dementia (Oh and Rabins, 2019). Late-onset AD, also known as the most common contributor of dementia (Van Cauwenberghe et al., 2016; Garcia-Blanco et al., 2017), is characterized by deposition of β-amyloid peptides and accumulation of intracellular neurofibrillary tangles (Morgan et al., 2019; van der Kant et al., 2020), which is followed by neuron death and a permanent loss of cognitive function (Wang et al., 2013; Minter et al., 2016; Lane et al., 2018). MCI, particularly amnestic MCI, often considered as an early phase of AD, is characterized by subtle impairment of memory and other cognitive functions (Petersen, 2004; Gauthier et al., 2006; Jicha et al., 2006), even though these symptoms have no obvious effects on daily living.

The pathological factors for AD are complex and heterogeneous. The incidence of AD increases with age. In addition to age, the most common cause of AD, genetic mutations are also risk factors known to confer AD susceptibility. Numerous genome-wide association studies (GWAS) on familial or sporadic AD have highlighted the hereditary and genetic predisposition of AD. For instance, mutations in *amyloid precursor protein* (*APP*) (Chartier-Harlin et al., 1991; Goate et al., 1991; Murrell et al., 1991), *presenilin 1* (*PSEN1*) (Mullan et al., 1992; Schellenberg et al., 1992; St George-Hyslop et al., 1992; Van Broeckhoven et al., 1992; Sherrington et al., 1995), and *PSEN2* genes (Levy-Lahad et al., 1995a; Levy-Lahad et al., 1995b; Rogaev et al., 1995) were the earliest identified genetic pathogenic factors causing familial AD. Subsequently, *apolipoprotein E* (*APOE*) gene ε4 and ε2 haplotypes were found associated with the risk of AD (Pericak-Vance et al., 1991; Corder et al., 1993; Strittmatter et al., 1993; Corder et al., 1994). Over the past decades, abundant GWAS works have identified that additional common or rare variants in multiple genes, such as *CLU*, *PICALM*, *CR1*, *MS4A4E*/*MS4A6A*, *CD2AP*, *CD33*, *EPHA1*, *ABCA7*, *SORL1*, *CASS4*, *CELF1*, *DSG2*, *FERMT2*, *HLA-DRB1*/*HLA-DRB5*, *INPP5D*, *MEF2C*, *NME8*, *PTK2B*, *SLC24A4*, *RIN3*, and *ZCWPW1* (Rogaeva et al., 2007; Lee et al., 2008; Harold et al., 2009; Lambert et al., 2009; Jun et al., 2010; Seshadri et al., 2010; Hollingworth et al., 2011; Naj et al., 2011; Reitz et al., 2011; Lambert et al., 2013), were genetically associated with AD. These AD susceptibility genes are involved in multiple pathways including synaptic cell endocytosis and functionality, hippocampal synapse function, amyloid pathway, Tau pathology, immunoinflammatory response, lipid metabolism and transport, cell migration, axonal transport and cytoskeletal function.

However, genetic studies on identical twins revealed incomplete concordance, thus implying that in addition to genetics, environmental and related factors also influence AD pathophysiology (Breitner et al., 1995; Raiha et al., 1996; Gatz et al., 1997; Pedersen et al., 2004). Moreover, genetic variants account only for approximately 5% of all AD patients, suggesting the possibility of epigenetic variations involved in the pathology of AD (Prasad and Jho, 2019). An increasing number of studies have confirmed the role of epigenetic factors in contributing to the etiopathology and progression of AD. One such pattern is DNA methylation, which reversibly regulates gene expression and interferes with the course of disease (Holliday and Pugh, 1975; Moore et al., 2013). DNA methylation has been successfully used as a biomarker for the diagnosis of other diseases, such as cardiopathy and cancer (Meder et al., 2017; Pan et al., 2018). Currently, many epigenome-wide association studies (EWAS) have investigated the differences of DNA methylome profiles in *postmortem* human brain tissue biospecimens between patients with AD and matched controls, which have identified several differentially methylated loci, such as *ANK1*, *BIN1*, *RPL13*, *CDH23* and *RHBDF2*, associated with cognitive decline and AD dementia progression (Bakulski et al., 2012; De Jager et al., 2014; Lord and Cruchaga, 2014; Lunnon et al., 2014; Watson et al., 2016; Ellison et al., 2017; Mano et al., 2017; Zhao et al., 2017; Gasparoni et al., 2018; Hernandez et al., 2018; Smith et al., 2018; Altuna et al., 2019; Fetahu et al., 2019; Lardenoije et al., 2019; Smith et al., 2019; Brokaw et al., 2020; Smith et al., 2021). Furthermore, additional genome-wide DNA methylation studies of peripheral blood in AD/dementia have also been performed and have revealed a number of AD-linked loci annotated to *NCAPH2*, *LMF2*, *B3GALT4* and *ZADH2* (Lunnon et al., 2014; Di Francesco et al., 2015; Kobayashi et al., 2016; Shinagawa et al., 2016; Madrid et al., 2018; Lardenoije et al., 2019; Vasanthakumar et al., 2020; Perez et al., 2022). Nevertheless, latent DNA methylation variation events occurs prior to detectable pathological hallmarks and visible clinical symptoms (Fransquet et al., 2020). To better understand the underlying changes of DNA methylation profiles in the early stages of AD, it is necessary to include MCI samples to identify possible signatures that can predict progression from a cognitively normal status to MCI, as well as from MCI to AD.

Numerous genome-wide methylation profiling studies of patients with AD from Caucasian populations have been performed over the past decades. However, little is known about the characteristics of DNA methylome profiles in white blood cells from MCI- and AD-affected Chinese patients, as well as the potential DNA methylation-based signatures associated with cognitive decline and AD trajectory. In this study, we revealed significant alterations in the DNA methylome profiles of blood leukocytes from Chinese patients with MCI and AD, in which several signature genes harboring differentially methylated positions might play a role in cognitive function recession and AD pathology. Furthermore, these findings indicated the presence of epigenetically dysregulated gene networks that might facilitate the development from cognitively normal state to MCI, and the subsequent transition from MCI to AD, in an integrally coordinated DNA methylation-dependent manner.

## 2 Materials and methods

### 2.1 Subject recruitment

The protocols of this study were reviewed and approved by the Ethics Committee of The Second People’s Hospital of Lishui (Zhejiang, China) before recruiting subjects (approval number: 20171116–1). Prior to participant enrollment, informed written consent was acquired from each subject or legal guardian of patient. The diagnostic criteria of the National Institute of Neurological and Communicative Disorders and Stroke-AD and Related Disorders Association (NINCDS-ADRDA) (McKhann et al., 1984) and the fourth edition of the Diagnostic and Statistical Manual of Mental Disorders (DSM-IV) was used for AD diagnosis. 20 patients with AD and 20 MCI subjects were recruited from Lishui City in Zhejiang province (China), from January 2019 to December 2019. In addition, 20 cognitively healthy subjects were enrolled as controls (Table 1). The brain of each AD patient was scanned by computed tomography (CT) and magnetic resonance imaging (MRI), and all AD-affected patients were diagnosed with brain atrophy. Individuals in the MCI group were recruited from the Department of Memory in The Second People’s Hospital of Lishui. MCI subjects were scored as 1.0 or 0.5 by the memory category of the Clinical Dementia Rating Scale (CDR) (Morris, 1993) or 0.5 by total CDR. All subjects from the MCI group showed memory problems without substantial impairment in diurnal living based on Petersen’s criteria of MCI (Petersen et al., 1999).

**TABLE 1:** Cohort demographics and clinical characteristics.

Parameters	CHCs	MCI	AD
Number	20	20	20
Gender (male/female)	3/17	10/10	4/16
Age (mean ± SD)	60.2 ± 5.3	65.7 ± 4.2	82.6 ± 8.4
MMSE score (mean ± SD)	28.8 ± 0.9	24.2 ± 2.6	6.9 ± 1.9
SMCI score (mean ± SD)	26.7 ± 0.5	20.5 ± 3.0	5.7 ± 2.0
AD8 score (mean ± SD)	0.4 ± 0.5	2.5 ± 0.6	7.6 ± 0.6
Obesity (BMI ≥30.0)	0	0	0
Smoking	0	0	0
Drinking	0	0	0
Mental illness	0	0	0
Diagnosed cancer	0	0	0
Diabetes mellitus	7	8	7
Hypertension	11	11	12
Coronary heart disease	8	6	7
Infectious conditions	0	0	0
Autoimmune diseases	0	0	0

Subject characteristics of 60 samples that passed QC. CHCs, denotes cognitively healthy controls; MCI, means mild cognitive impairment; AD, means Alzheimer’s disease; SD, denotes standard deviation; BMI, means body mass index (kg/m^2^); MMSS, means Mini-Mental State Examination; SMCI, means Screening Scale for Mild Cognitive Impairment; AD8, means Alzheimer’s Disease-8.

The Chinese versions of Mini-Mental State Examination (MMSE), Screening Scale for Mild Cognitive Impairment (SMCI) and Alzheimer’s Disease-8 (AD8) were used to score the cognitive and functional status of all subjects. Demographic data and detailed clinical information for all participants are shown in Table 1. The exclusion criteria were as follows: early-onset AD or familial AD dementia; another type of neurodegenerative or mental disorders such as Parkinson’s disease, depression and schizophrenia; Diagnosed cancer/tumor such as gastric carcinoma and breast cancer; other autoimmune or inflammatory diseases such as inflammatory bowel condition; active infectious diseases included bacterial, fungal, or viral infections; obesity (body mass index ≥30.0); smoking and drinking.

### 2.2 Genome-wide DNA methylation analysis

Genomic DNA was extracted from peripheral white blood cells of 20 patients with AD, 20 patients with MCI and 20 healthy controls using Blood DNA Extraction Kit (QIAGEN) according to the manufacturer’s protocol, which was followed by bisulfite conversion using the EZ DNA Methylation-Direct Kit (Zymo Research, Orange, CA) and processed according to Illumina protocols. Subsequently, converted DNA was scanned with the Infinium MethylationEPIC array BeadChips (850 K chip) (Illumina Inc., California, United States). All 60 samples were processed together to minimize batch effects.

### 2.3 Quality control and data processing

An overview of the methodological flow in this study is shown in the Supplementary Figure S1. Data analysis of raw intensity (.idat file) was performed in R software using the ChAMP pipeline (Morris et al., 2014; Tian et al., 2017) from Bioconductor with default settings. The quality control (QC) analysis started by screening samples. Samples of technical replication and from smokers, as well as samples with a *p*-value >0.01 in at least 5% probes, were removed from further analysis. BeadArray Controls Reporter software (https://support.illumina.com/downloads/beadarray-controls-reporter-installer.html) was used to perform experimental quality control, and no one sample with low experimental quality was excluded. Gender status was examined by adopting the “minfi” R package (Teschendorff et al., 2009). Background correction and dye-bias normalization were conducted by using the ChAMP.norm function. The initial step involved removal of low-quality probes. Probes with a detection *p*-value >0.01 in more than 5% samples, or with bead count <3 in at least 5% of samples, were removed. This step led to the removal of 3,973 and 7,642 probes with poor quality, respectively. Then probes related to SNPs (n = 96,381) or probes aligned to multiple locations (n = 11) were removed, as well as probes for non CpG sites (n = 2,972). Furthermore, probes on X and Y chromosomes (n = 16,697) were removed to avoid any deviation caused by gender differences. The methylation ratios of a certain CpG site were represented by beta values ranging from 0 to 1.0. The beta mixture quartile (BMIQ) method implemented in the ChAMP R package was used to adjust beta values. Significant variation (*p*-value <0.05) arising from the slide variable were completely removed after running the ComBat program. Following the standard procedures QC procedures described above, 722,324 probes across 60 samples, including 20 patients with AD, 20 MCI subjects and 20 unaffected controls, were available for subsequent analysis.

### 2.4 Cell type correction

Cell percentage differences between heterogeneous samples, such as blood cells, between patients and unaffected controls needed to be examined and controlled in DNA methylation analysis. Cell type heterogeneity was adjusted by using the ChAMP.refbase function.

### 2.5 Differentially methylated position analysis

The champ. SVD () function (Teschendorff et al., 2009) was used to investigate the effects of age and sex, and the champ. runCombat () method was used to adjust age and sex confounders. An adjusted beta value matrix was used to identify differentially methylated positions (DMPs) by using the ChAMP.DMP function. DMPs were determined by comparing the beta values per single nucleotide at each cytosine ‘CpG’ locus between MCI subjects or patients with AD and cognitively healthy controls, as well as patients with AD and MCI subjects. *p* values were adjusted by the Benjamini–Hochberg (BH) procedure. Probes with an adjusted *P* (*Padj*) value <0.05 were considered significant. Bonferroni correction was used to perform multiple testing with a *p*-value <6.68 × 10^−8^ (corresponding to *Padj* value <0.05) as the significance threshold. Epigenome-wide association studies (EWAS) were performed to identify MCI- and AD-associated DMPs after regressing age and gender. EWAS was carried out by a logistic regression model implemented in GLINT (Rahmani et al., 2017).

### 2.6 Differentially methylated region analysis

Differentially methylated regions (DMRs) combine methylation information from multiple neighboring CpG sites, were identified by using the function ChAMP.DMR with the Lasso method (Butcher and Beck, 2015). For each DMR, a minimum number of three consecutive CpG sites and the distance between two adjacent DMPs less than 1,000 bp were required to constitute an individual DMR. Regions with a *Padj* value <0.05 corrected by BH method were considered significant. All DMRs were annotated by using ChAMP.import function.

### 2.7 Bioinformatic analysis

Unsupervised principal component analysis (PCA) was performed by using prcomp function in R. Supervised analysis such as partial least squares-discriminant analysis (PLS-DA) was performed by the R package mixOmics (Rohart et al., 2017). A Manhattan plot was created using the R package qqman. Gene Ontology (GO) and Kyoto Encyclopedia of Genes and Genomes (KEGG) enrichment analysis was performed by using R package clusterProfiler (Yu et al., 2012). Volcano plots, bar plots, pie charts and violin plots were created by the R package ggplot2. Protein-protein interaction (PPI) analysis was performed by online tool STRING (www.string-db.org/) (Szklarczyk et al., 2021).

### 2.8 Receiver operating characteristic curve analysis of combined DMPs

Ten samples from each group were selected randomly as the training data set. The rest of samples were combined as the validation data set. The R function glm was used to create linear models. Receiver operating characteristic (ROC) curve analysis was performed using pROC in R package (Robin et al., 2011), and ggplot2 was used to plot ROC curves to identify the performance of models.

### 2.9 Tissue enrichment and transcription factor motif enrichment analysis

Tissue enrichment analysis was performed by online TissueEnrich tools (https://tissueenrich.gdcb.iastate.edu/) (Jain and Tuteja, 2019). Human protein atlas database was used as data set (Yu N. Y. et al., 2015). Transcription factor motif enrichment analysis was performed with the AME online tool (https://meme-suite.org/meme/tools/ame) (McLeay and Bailey, 2010; Bailey et al., 2015). The 100 bp upstream and downstream sequences of target probe were used as input. The HOCOMOCO Human (v11 CORE) was used as motif database.

### 2.10 Statistical analyses

Bonferroni correction was used to perform multiple testing adjustment in EWAS. Benjamini–Hochberg approach was used for correction to obtain *Padj* value. Independent *t*-tests, Mann-Whitney *U*-tests, and White’s non-parametric *t*-tests were used to analyze continuous variables. Statistical analysis was performed with SPSS V19.0 software (Chicago, IL, United States). Statistical significance was tested using two-sided approach, and only a *p*-value <0.05 or a corrected *Padj* value <0.05 was considered statistically significant.

## 3 Results

### 3.1 Identification of differentially methylated positions and regions

**FIGURE 1:**
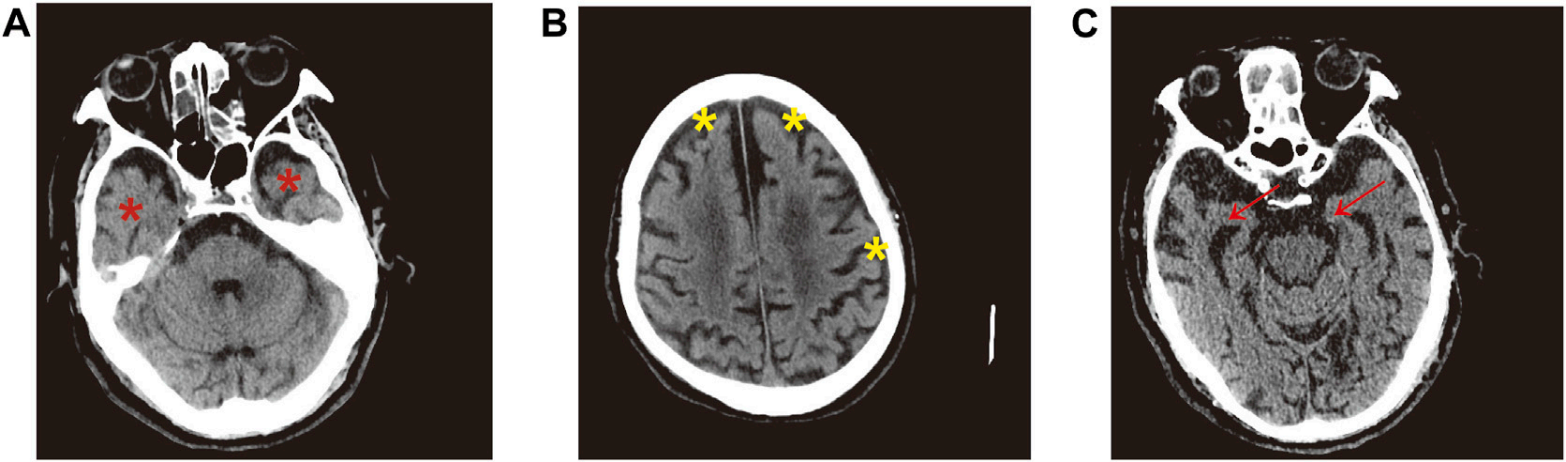
Representative brain CT images of AD patients. **(A)** Red asterisk indicates obvious atrophy of temporal lobe and dilated temporal horn in the bilateral brain. **(B)** Significantly atrophied cerebral gyrus, yellow asterisk denotes deepened sulcus. **(C)** Red arrow represents distinctly reduced transverse diameter of hippocampus in the bilateral brain.

The demographic information and clinical characteristics of all subjects are shown in Table 1. In total, 20 AD-affected patients, 20 subjects with MCI and 20 cognitively healthy controls (CHCs) were enrolled. There were significant differences in age range and gender ratio between the three groups (*p* < 0.05); these differences were controlled well in this study. The results of singular value decomposition analysis suggested that age had no effect on the principal component (PC)-1∼8, and that sex had only a mild effect on the PC-7 (Supplementary Figure S2). In contrast, the sample group had a major effect on PC-1, and PC-3∼6, and slide had a significant effect on PC-1 and PC-5 (Supplementary Figure S2), thus implying that AD and MCI disease status and batch, but not age and sex, had dominant effects on our data. Despite this, we adjusted the age and sex along with slide confounders to exclude their effects. Participants who smoked, drank alcohol or were obese, as well as subjects diagnosed with a concurrent mental illness, cancer, autoimmune and infectious diseases, were excluded from subsequent analysis. No significant differences were observed in the number of subjects with chronic diseases such as hypertension, diabetes mellitus and coronary heart disease (*p* > 0.05). The MMSE and SMCI scores for patients with AD and MCI were significantly lower than that of CHCs (*p* < 0.05) while the AD8 score was significantly higher in patients with MCI and AD than in CHCs (*p* < 0.05), thus suggesting impaired cognitive function and severe dementia status in the MCI and AD patients. Moreover, brain computed tomography results showed that all AD patients exhibited typical AD symptoms, such as obvious atrophy of the temporal lobe and the cerebral gyrus, dilated temporal horn, deepened sulcus, and reduced transverse diameter of the hippocampus (Figures 1A–C), thus certifying that all of the patients recruited were definitely clinically diagnosed with AD.

To identify the overall changes in DNA methylation levels of peripheral blood leukocytes in Chinese patients with MCI and AD, we extracted genomic DNA from the blood leukocytes of 20 patients with MCI, 20 patients with AD and 20 CHC samples. These samples were analyzed with Infinium Methylation EPIC array BeadChips which features probes for more than 850,000 CpGs per sample. The results of both unsupervised PCA and supervised analysis such as PLS-DA, showed a good separation of AD and MCI samples from CHCs (Supplementary Figures S3A, B), thus suggesting a significant change in the methylome profiles of peripheral leukocytes from Chinese patients with AD and MCI.

**FIGURE 2:**
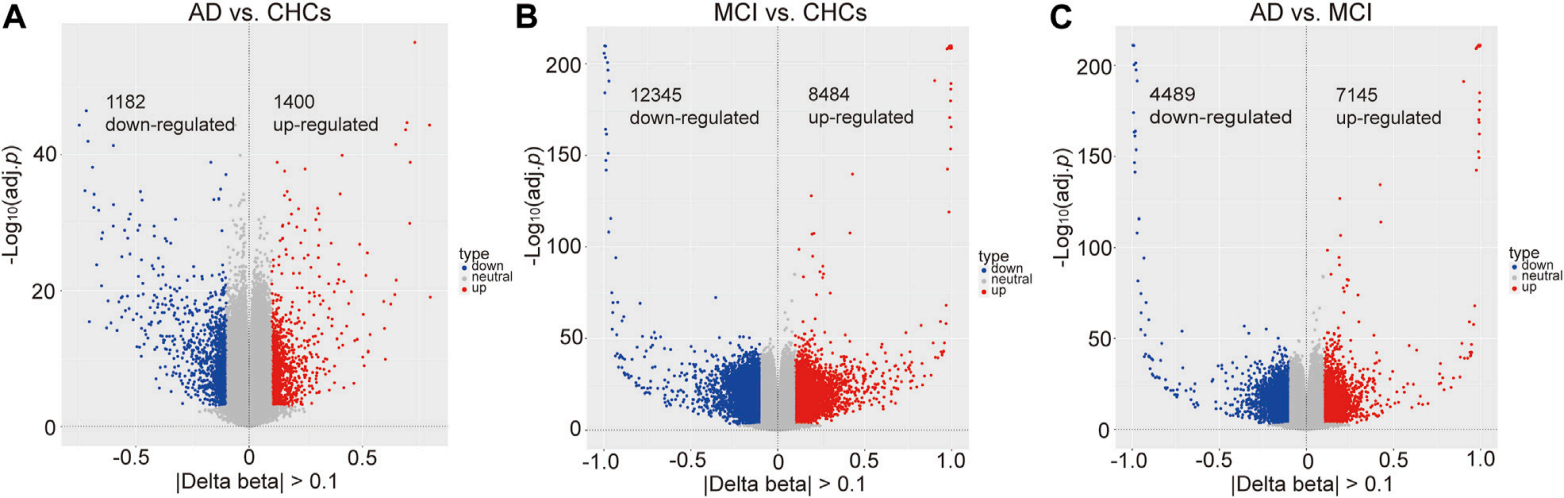
Significant differential methylated positions between AD, MCI and cognitively healthy controls. **(A)** Differentially methylated positions between AD and cognitively healthy controls (CHCs), *Padj* value <0.05, |Delta beta| cutoff >0.1. 1400 hypermethylated and 1182 hypomethylated positions. **(B)** Differentially methylated positions between MCI and CHCs, *Padj* value <0.05, |Delta beta| cutoff >0.1. 8,484 hypermethylated and 12,345 hypomethylated positions. **(C)** Differentially methylated positions between AD and MCI, *Padj* value <0.05, |Delta beta| cutoff >0.1. 7,145 hypermethylated and 4,489 hypomethylated positions.

A total of 722,324 CpG sites passed standard quality control procedures; of these, 184,316 and 381,573 probes had a *Padj* value <0.05 (Bonferroni correction) for AD *versus* CHCs and MCI *versus* CHCs (Supplementary Figures S4A, B), respectively, thus highlighting distinct differences in global DNA methylation profiles between AD patients or MCI subjects and CHCs. Furthermore, 265,194 probes passed Bonferroni adjustment for AD *versus* MCI (*Padj* < 0.05) (Supplementary Figure S4C). When |Delta beta| thresholds were considered by volcano plots, compared to CHCs, 1,400 significantly hypermethylated and 1,182 significantly hypomethylated DMPs were identified in AD samples (*Padj* < 0.05, |Delta beta| > 0.1) (Figure 2A; Supplementary Table S1). In addition, 8,484 significantly hypermethylated and 12,345 significantly hypomethylated DMPs were identified in MCI *versus* CHCs (*Padj* < 0.05, |Delta beta| > 0.1) (Figure 2B; Supplementary Table S2). Moreover, 7,145 significantly hypermethylated and 4,489 significantly hypomethylated DMPs were identified in AD *versus* MCI (*Padj* < 0.05, |Delta beta| > 0.1) (Figure 2C; Supplementary Table S3). When the |Delta beta| cutoff was set at 0.2, 272 significantly hypermethylated and 179 significantly hypomethylated DMPs were observed in AD *versus* CHCs (Supplementary Figure S5A; Supplementary Table S4). In addition, 1,100 significantly hypermethylated and 1,327 significantly hypomethylated DMPs were identified in MCI *versus* CHCs (Supplementary Figure S5B; Supplementary Table S5). Furthermore, 574 significantly hypermethylated and 439 significantly hypomethylated DMPs were identified in AD *versus* MCI (Supplementary Figure S5C; Supplementary Table S6). In addition, significant DMRs were identified by combining signals from nearby CpG positions in the three comparative groups. Compared with CHCs, 142 and 3,831 significant DMRs were identified in AD and MCI samples (Supplementary Tables S7, S8), overlapping with 119 and 2,716 unique genes, respectively; Furthermore, 1,131 significant DMRs were identified in AD patients when compared to MCI subjects, overlapping with 911 unique genes (Supplementary Table S9). Furthermore, 45 unique genes (1.23%) were shared by the three comparative groups.

**FIGURE 3:**
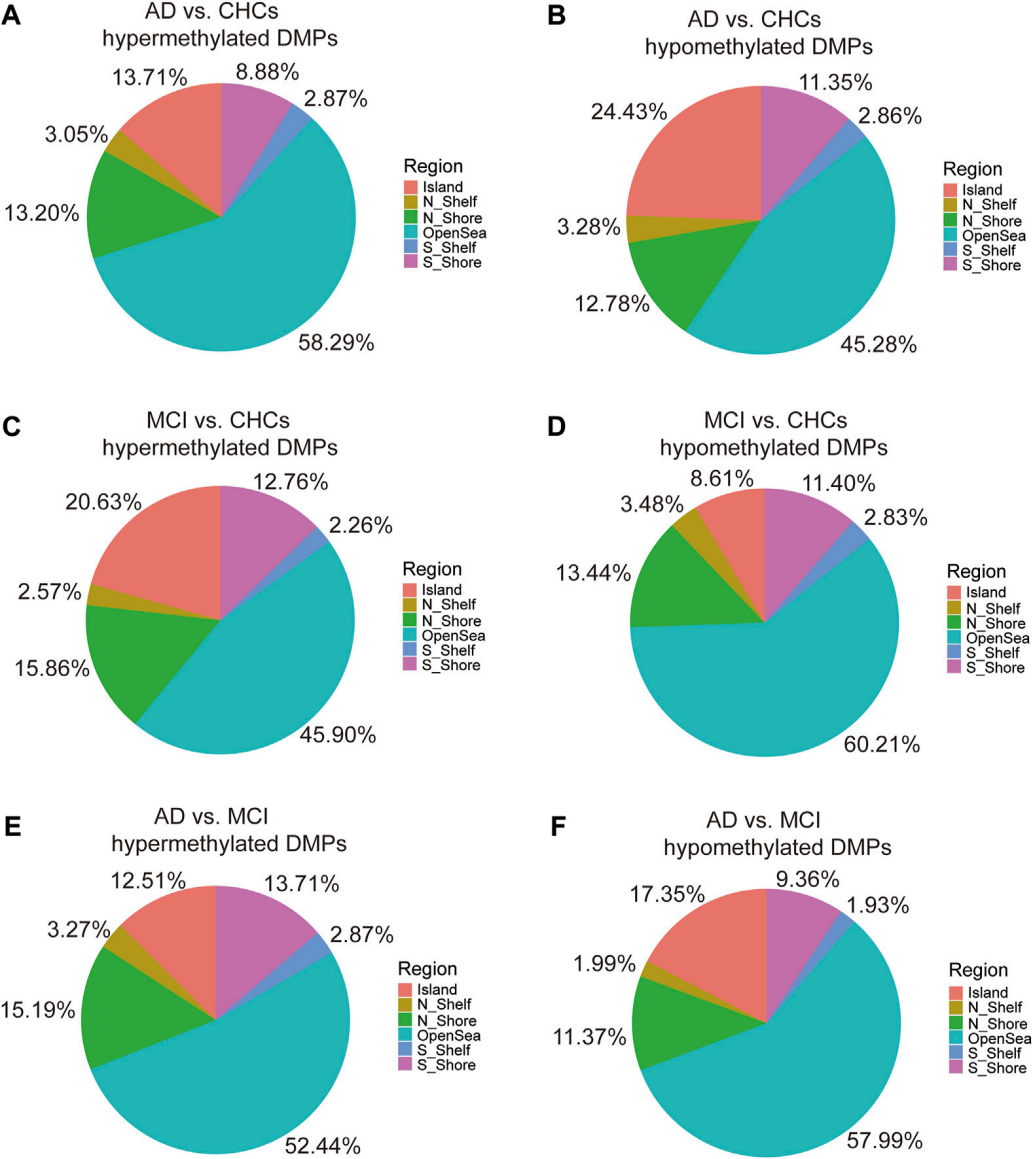
Pie chart indicating the location of DMPs relative to CpG islands. **(A, B)** The percentage of various CpG island locations harboring significant (*Padj* value <0.05) hypermethylated and hypomethylated positions between the AD and CHCs. **(C, D)** The percentage of various CpG island locations harboring significant (*Padj* value <0.05) hypermethylated and hypomethylated positions between the MCI and CHCs. **(E, F)** The percentage of various CpG island locations harboring significant (*Padj* value <0.05) hypermethylated and hypomethylated positions between the AD and MCI groups. Domains are labeled with different colors. N_Shelf, 2–4 kb upstream of CpG island; N_Shore, 0–2 kb upstream of CpG island; OpenSea, >4 kb from a CpG island; S_Shelf, 2–4 kb downstream of CpG island; S_Shore, 0–2 kb downstream of CpG island.

Functional genomic regions of the significant DMPs are shown in Supplementary Figure S6. In AD *versus* CHCs, the majority of hypermethylated and hypomethylated DMPs were located in gene bodies and regulatory regions, including body, first Exon, 3′ UTR, 5′ UTR, TSS200, and TSS1500, while relatively fewer DMPs were aligned to other regions (Supplementary Figures S6A, B). A similar distribution pattern was also observed in MCI *versus* CHCs and AD *versus* MCI (Supplementary Figures S6C–F). Furthermore, the majority of hypermethylated DMPs were scattered in open sea areas (located >4.0 kb from a CpG island), while the minority of hypermethylated DMPs were found near or within CpG islands in AD *versus* CHCs (Figure 3A). However, an opposite pattern was shown with hypomethylated DMPs from AD *versus* CHCs (Figure 3B). Unlike AD *versus* CHCs, an opposite pattern was seen in the hypermethylated and hypomethylated DMPs from MCI *versus* CHCs (Figures 3C, D). Furthermore, most hypermethylated and hypomethylated DMPs were scattered in open sea areas, whereas fewer DMPs were near or within CpG islands in the AD *versus* MCI (Figures 3E, F). Ternary plots also showed the same results (Supplementary Figure S7).

### 3.2 Potential DNA methylation biomarkers related to AD

**FIGURE 4:**
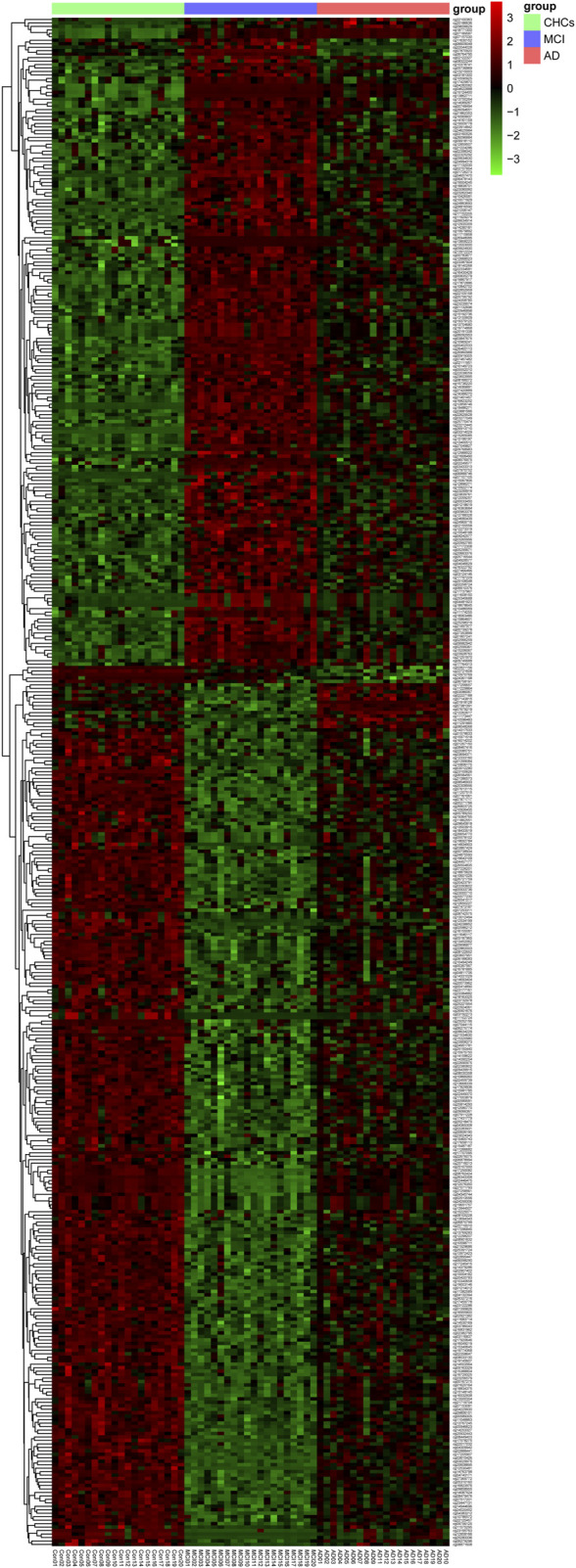
Heatmap of the common 441 DMPs. Total of 441 significant DMPs (*Padj* value <0.05) were overlapped by the three comparative groups, i.e., AD *versus* CHCs, MCI *versus* CHCs, and AD *versus* MCI. Red and green denote upregulated and downregulated, respectively. AD group: AD 01–20, MCI group: MCI 01–20, CHCs group: Con 01–20. The heatmap was plotted using the Pheatmap package in R (v.3.3.2).

**FIGURE 5:**
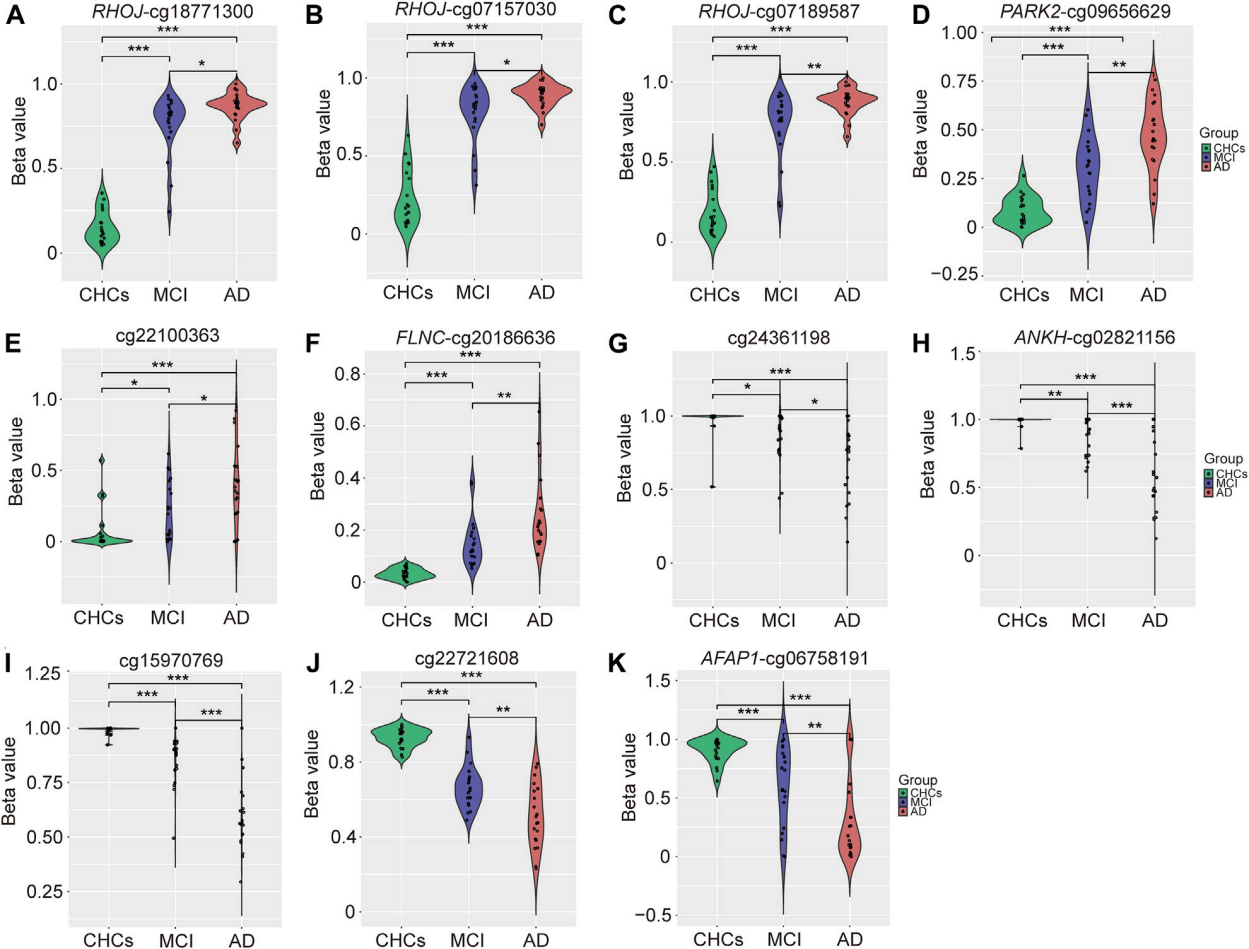
Violin plots of continuously hyper- and hypomethylated CpG sites in MCI and AD. **(A–F)** 6 continuously hypermethylated CpGs in MCI and AD compared to CHCs. **(G–K)** 5 continuously hypomethylated CpGs in MCI and AD relative to CHCs. Patients with AD and MCI, and CHCs are labeled with red, purple, and green, respectively. The *Y*-axis represents the beta value of each CpG site. BH procedure was used for correction to obtain *Padj* value. *: *Padj* < 0.05, **: *Padj* < 0.01, ***: *Padj* < 0.001.

**FIGURE 6:**
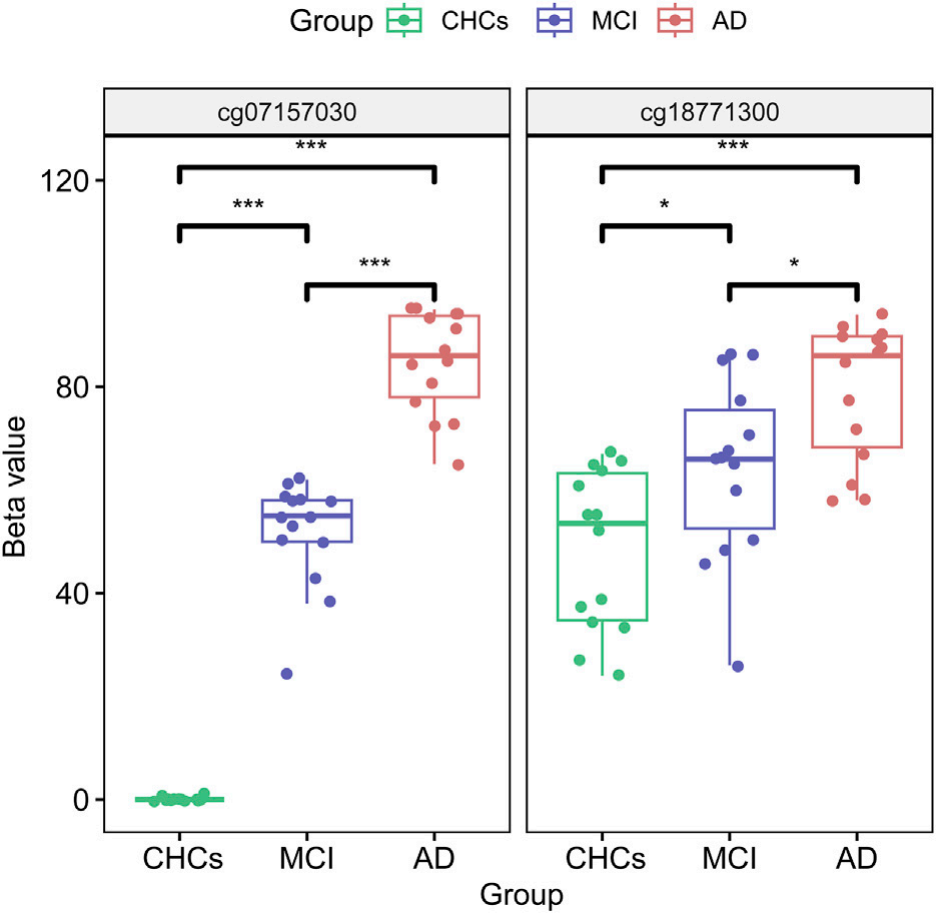
cg07157030 and cg18771300 were increasingly methylated in MCI and AD. The two DMPs of cg07157030 and cg18771300 were validated by pyrosequencing in independent samples of MCI, AD and CHCs. The BH procedure was used for correction to obtain a *Padj* value. *: *Padj* < 0.05, ***: *Padj* < 0.001.

To identify potential DMPs biomarkers related to cognitive impairment and AD, we mainly focused on the common DMPs between the three comparative groups of AD *versus* CHCs, MCI *versus* CHCs, and AD *versus* MCI. A total of 441 common DMPs aligning to 213 unique genes were identified (Figure 4; Supplementary Figures S8A, B; Supplementary Table S10). Of these common DMPs, 6 CpG sites were continuously and significantly hypermethylated (i.e., DNA methylation level: AD > MCI > CHCs) in MCI and AD samples compared to CHCs (*Padj* < 0.05), including *RHOJ* cg18771300, *RHOJ* cg07157030, *RHOJ* cg07189587, *PARK2* cg09656629, cg22100363, and *FLNC* cg20186636 (Figures 5A–F), while 5 CpG sites were continuously and significantly hypomethylated (i.e., DNA methylation level CHCs > MCI > AD) in MCI and AD groups relative to CHCs (*Padj* < 0.05), such as cg24361198, *ANKH* cg02821156, cg15970769, cg22721608, and *AFAP1* cg06758191 (Figures 5G–K). To further exclude the effects of age and sex confounders, the analysis of age and sex adjustment was performed. The results showed that the correction of age and sex had no dramatic influences on the identified significant DMPs, such as *FLNC* cg20186636 (Supplementary Figure S9). Pyrosequencing results further demonstrated that cg07157030 and cg18771300 were significantly and increasingly methylated in independent samples of MCI and AD when compared to that of CHCs (*Padj* < 0.05) (Figure 6).

**FIGURE 7:**
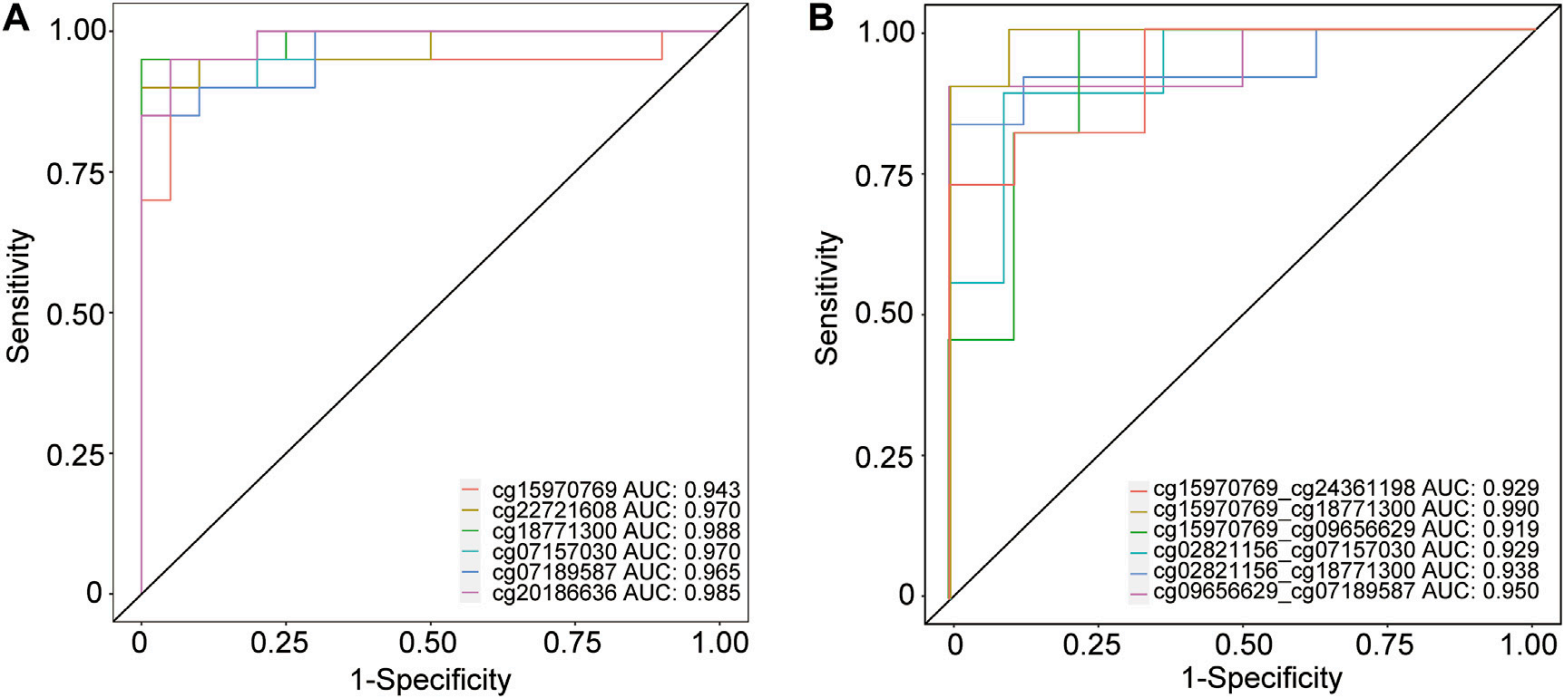
ROC analysis of several CpG position for predicting MCI and AD events. **(A)** Receiver operating characteristic (ROC) curves analysis of 6 DMPs including cg15970769, cg22721608, cg18771300, cg07157030, cg07189587, and cg20186636 in predicting the transition of CHCs to MCI. **(B)** ROC analysis of several combinations of two CpG sites, such as cg15970769 and cg24361198, cg15970769 and cg18771300, cg15970769 and cg09656629, cg02821156 and cg07157030, cg02821156 and cg18771300, and cg09656629 and cg07189587, in predicting MCI and AD conversion. ROC analysis was performed using pROC in the R package.

The results of ROC analysis showed that several DMPs had an area under the curve (AUC) > 0.900 in predicting MCI event (Figure 7A), for instance, cg18771300 (AUC = 0.988, confidence interval:0.955–1.000, specificity = 100.00%, sensitivity = 95.00%), cg20186636 (AUC = 0.985, confidence interval:0.950–1.000, specificity = 95.00%, sensitivity = 95.00%), cg22721608 (AUC = 0.970, confidence interval:0.910–1.000, specificity = 100.00%, sensitivity = 90.00%), cg07157030 (AUC = 0.970, confidence interval:0.915–1.000, specificity = 100.00%, sensitivity = 85.00%), cg07189587 (AUC = 0.965, confidence interval:0.900–1.000, specificity = 100.00%, sensitivity = 85.00%) and cg15970769 (AUC = 0.943, confidence interval:0.838–1.000, specificity = 95.00%, sensitivity = 95.00%) (Supplementary Table S11). In addition, several combinations of two DMPs had an AUC >0.9000 in predicting MCI and AD conversion, such as cg15970769 and cg18771300 (AUC = 0.990, confidence interval:0.940–1.000, specificity = 90.00%, sensitivity = 100.00%), cg09656629 and cg07189587 (AUC = 0.950, confidence interval:0.820–1.000, specificity = 100.00%, sensitivity = 90.00%), cg02821156 and cg18771300 (AUC = 0.938, confidence interval:0.792–1.000, specificity = 100.00%, sensitivity = 83.33%), cg15970769 and cg24361198 (AUC = 0.929, confidence interval:0.798–1.000, specificity = 88.89%, sensitivity = 81.82%), cg02821156 and cg07157030 (AUC = 0.929, confidence interval:0.778–1.000, specificity = 90.91%, sensitivity = 88.89%), as well as cg15970769 and cg09656629 (AUC = 0.919, confidence interval:0.758–1.000, specificity = 77.78%, sensitivity = 100.00%) (Figure 7B). A single DMP had reduced potential for predicting the transition of MCI to AD (Supplementary Figure S10). In addition, another combination of DMPs also showed potential ability for predicting MCI and AD events (Supplementary Table S11). Collectively, these data suggested that these combinations of significant DMPs have the potential to act as biomarkers for the prediction of AD onset and progression.

### 3.3 Functional enrichment and tissue-specific expression analysis of common differentially methylated genes

**FIGURE 8:**
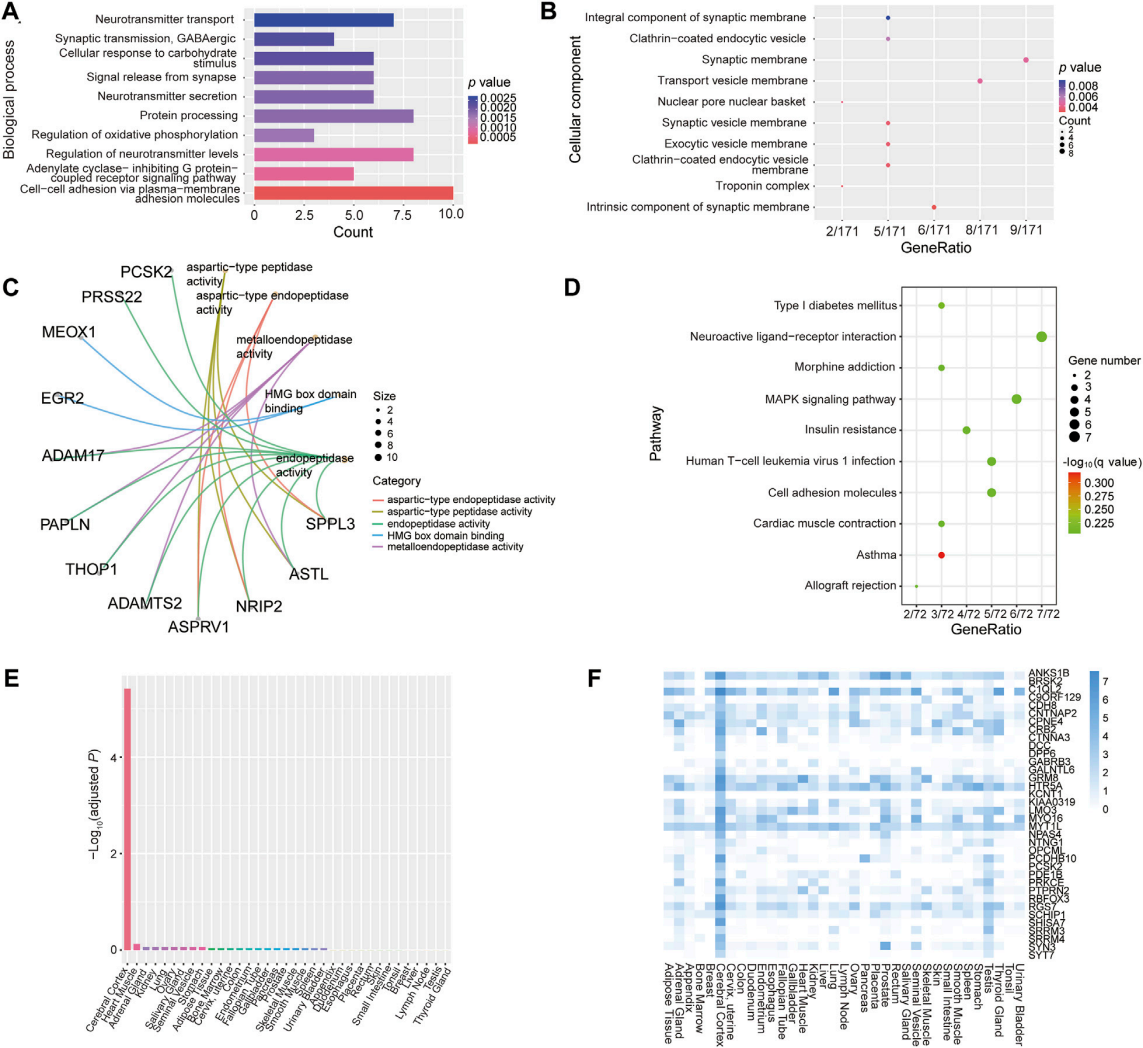
Functional enrichment analysis of epigenetically dysregulated genes harboring the common 441 DMPs. **(A–C)** Gene ontology enrichment analysis results of the 213 unique genes harboring the common 441 DMPs. **(A–C)** represents enrichment analysis of biological process, cellular component and molecular function, respectively. **(D)** KEGG pathway enrichment analysis results. **(E, F)** Tissue-specific expression enrichment analysis of the overlapping 213 genes, which was performed by using online TissueEnrich tools.

To further investigate the function of epigenetically dysregulated genes harboring the common 441 DMPs, we performed GO and KEGG pathway enrichment analysis, as well as PPI and tissue expression enrichment analyses. GO annotation results showed that these overlapping genes were mainly enriched in multiple biological processes of neural activities, such as neurotransmitter transport, GABAergic synaptic transmission, signal release from synapse, neurotransmitter secretion, and regulation of neurotransmitter levels (Figure 8A). Furthermore, the proteins encoded by these epigenetically dysregulated genes were primarily localized in synaptic membrane, synaptic vesicle membrane, and transport vesicle membrane (Figure 8B). The main function of these common genes was aspartic type/metallo-endopeptidase activity (Figure 8C). In addition, these epigenetically dysregulated genes involved in multiple pathways, including neuroactive ligand-receptor interaction, MAPK signaling, and cell adhesion (Figure 8D). Moreover, TissueEnrich tools analysis showed identified 36 tissue-specific overlapping genes, which encoded proteins that were enriched significantly in the cerebral cortex (Fold change = 2.58, -Log_10_ *Padj* = 5.42), such as SYT7, SYN3, and KCNT1 (Figures 8E, F; Supplementary Table S12). The results of PPI analysis revealed that the proteins encoded by these common genes constitute complex and tight networks (Supplementary Figure S11). Taken together, these epigenetically dysregulated genes might play an important role in cognitive impairment and AD etiology.

It was well known that differential methylation can occur at specific sites (Hannon et al., 2018). To explore the underlying transcription factor binding motif around the common 441 DMPs, we performed enrichment analysis of the transcription factor binding motif. All of the significantly enriched transcription factors and their binding motifs are shown in Supplementary Figure S12. As expected, some identified transcriptional factors recognize and bind to purine- or GC-rich motifs among the common 213 genes. For instance, KLF12, KLF10, as well as SP2 and SP4 bind to GC-rich motifs, while ETV1 and ETV6 recognize purine-rich sequences (Supplementary Figure S12).

## 4 Discussion

Epigenetic mechanisms such as DNA methylation, play an important role in the etiopathology of neurodegenerative disorders including AD, dementia with Lewy bodies, vascular dementia, Parkinson’s disease and AD-like conditions (Fransquet et al., 2018; Fransquet and Ryan, 2019; Li et al., 2019). Previous study suggested that various neurodegenerative diseases shared similar aberrant DNA methylation pattern in certain gene set (Sanchez-Mut et al., 2016). However, objective and reliable biomarkers for the early diagnosis of AD dementia are still absent (Zetterberg and Burnham, 2019), which would impede the decisions about effective prevention and timely interventions before the appearance of clinical symptoms (Huang et al., 2020).

There were some studies suggested DNA methylation levels reduced in certain brain region from patients with AD or suffering aging compared to controls (Wang et al., 2008; Mastroeni et al., 2009; Mastroeni et al., 2010; Hernandez et al., 2011; Mastroeni et al., 2011; Chouliaras et al., 2013), whereas other studies indicated the level of DNA methylation was increased in AD patients relative to healthy controls (Bollati et al., 2011; Coppieters et al., 2014; Di Francesco et al., 2015). DNA methylation within the brain plays a critical role in memory (Miller and Sweatt, 2007; Miller et al., 2010). Also, DNA methylation has been suggested play an indispensable role in the etiopathology of AD (Mastroeni et al., 2010; Tong et al., 2015). Aberrant epigenetic changes in CpG islands may enhance the pathology of late-onset AD (Wang et al., 2008). A number of studies indicated that amyloidogenic pathway-involved genes, such as amyloid precursor protein (*APP*) (West et al., 1995; Tohgi et al., 1999; Wang et al., 2008; Barrachina and Ferrer, 2009; Hou et al., 2013; Iwata et al., 2014), β-secretase 1 (*BACE1*) (Do Carmo et al., 2016; Li et al., 2019), presenilin 1 (*PSEN1*) (Fuso et al., 2005; Fuso et al., 2008), sortilin-related receptor 1 (*SORL1*) (Scherzer et al., 2004; Yu L. et al., 2015), and neprilysin (*NEP*) (Chen et al., 2009; Nagata et al., 2018), were differentially methylated in AD patients or in animal model of AD compared to that of controls, even though current therapeutic strategies targeting Aβ are not satisfactory in AD treatment (Panza et al., 2019). Interestingly, one recent study revealed that aberrant autolysosome acidification-induced autophagy barrier, but not amyloidogenic pathway, was likely to be the most fundamental causal factor for AD (Lee et al., 2022), which might provide us with a more promising therapeutic strategy against AD onset and progression. Furthermore, a previous study showed that abnormal methylation in circadian genes such as *CRY1* and *PER1* leads to dementia symptoms (Liu et al., 2008). Moreover, one recent epigenome-wide association study identified that *HOXB6* gene was robustly hypermethylated in MCI and AD blood samples compared with healthy controls (Roubroeks et al., 2020), suggesting *HOXB6* gene hypermethylation signature may be potential biomarker for the diagnosis of MCI and AD.

In this study, we assessed the global changes of leukocyte DNA methylation in MCI- and AD-affected Chinese patients compared to that of cognitively healthy controls and reported multiple potential DNA methylation-based signatures associated with cognitive decline and AD, including CpG positions harbored in *RHOJ*, *PARK2*, *FLNC*, *ANKH*, and *AFAP1* genes. A previous study suggested that the Ras homolog (Rho) kinase pathway was changed in leukocytes and the brains of subjects with Huntington’s Disease (Narayanan et al., 2016). Ras homolog gene family member A (RhoA) involved in vascular dementia and serves as potential targets of new drugs for vascular dementia treatment (Wang et al., 2018). Furthermore, a role of aberrant RhoA signaling involved in multiple neurodegenerative disease such as AD, Parkinson’s disease, and Huntington’s disease (Schmidt et al., 2022). Fasudil, the first clinically administered inhibitor of Ras homolog-associated kinase, and is currently used as a therapeutic target for neurodegenerative disorders (Wang et al., 2022), suggesting that drugs targeting Rho/Rho-associated kinases have the potential to alleviate neurodegenerative conditions such as AD. In this study, we found that the methylation level of three significant CpG sites, including cg18771300, cg07189587 and cg07157030 within *Ras homology family member J* (*RHOJ*) gene, was significantly elevated in MCI and AD samples compared with cognitively healthy controls (*Padj* < 0.05). These findings proposed a potential role of *RHOJ* in epigenetic regulation of cognitive impairment and AD etiopathology. The three remarkedly hypermethylated CpG sites, includes cg18771300, cg07189587 and cg07157030, may be able to serve as reliable biomarkers to predict AD onset and progression. Furthermore, *RHOJ* might have the potency to be developed as a potential therapeutic target against AD progression in the future.

It is well known that cognitive decline and AD dementia are highly complex conditions, which is caused by both genetic and environmental factors (Kivipelto et al., 2018; Lourida et al., 2019). Apart from ethnic background, environmental factors such as geographical conditions, lifestyle and dietary habits, can also elicit epigenetic alterations like DNA methylation changes that have been manifested to be related with dementia both in peripheral blood and in brain (Fransquet et al., 2018). Therefore, the epigenome landscape of AD-affected Chinese patients is likely to differ from that of Caucasian population with AD. Indeed, our study found that the DNA methylome of peripheral blood cells from Chinese patients with AD was significantly different from those of AD-affected Caucasian patients. Previous studies have highlighted the association of *ANK1* and *BIN1* methylation changes with AD dementia neuropathology in a Caucasian population (De Jager et al., 2014; Lunnon et al., 2014; Chibnik et al., 2015; Watson et al., 2016; Salcedo-Tacuma et al., 2019; Semick et al., 2019). In particular, altered DNA methylation has been shown to regulate the expression of *BIN1* (Wechsler-Reya et al., 1997), and several studies have suggested that *BIN1* expression was changed in the AD brain (Chapuis et al., 2013; Glennon et al., 2013; De Rossi et al., 2016). Another study on Chinese patients with AD suggested that *UQCRC1* was hypermethylated in AD-affected patients relative to healthy controls and found that *UQCRC1* hypermethylation was notably associated with the expression levels of *CTSB*, *CTSD*, *DDT* and *NRD1* (Ma et al., 2016). In the current study, we identified the most significant DMPs (*Padj* < 0.05), including cg18771300, cg07157030, cg07189587, cg09656629, cg20186636, cg02821156, and cg06758191, in Chinese patients with AD relative to cognitively healthy controls, which aligned to *RHOJ*, *PARK2*, *FLNC*, *ANKH*, and *AFAP1* genes (Figure 5), respectively. These significant DMPs was continuously hypermethylated or hypomethylated in MCI and AD compared with cognitively healthy controls, implying that these DNA methylation-based signatures have the potential as a biomarker for MCI and AD diagnosis. The inconsistent results from different AD methylome studies probably due to multiple possible factors, for example, distinct ethic background (Caucasian or Chinese population), the type of tissue collected (*postmortem* human brain tissue or blood cell), sampling time point during disease course (early or late stage), different sample sizes, as well as various environmental factors.

However, our current study had several limitations. First, the sample size of patients with MCI and AD, as well as non-dementia controls, was relatively small, which might give rise to inaccurate results. Given that the small sample size would limit the power to detect differentially methylated sites and dysregulated genes (Zhang et al., 2020), we now need to perform a validation assay on an independent sample cohort in our further studies. Second, the age range and sex ratio of all participants recruited in this study were very different when compared between the three comparative groups, thus creating more difficulty when analyzing the methylome data. To further exclude the effects of age and sex, we adjusted age and sex confounders when identifying DMPs, even though the results of singular value decomposition analysis suggested that age had no effect on the principal component (PC)-1∼8, and sex had only a mild effect on the PC-7 (Supplementary Figure S2). Unlike age and sex confounders, the sample group had a major effect on PC-1, and PC-3∼6; moreover, slide confounders had a major effect on PC-1 and PC-5 (Supplementary Figure S2), thus implying AD and MCI disease status and batch, but not age and gender, have dominant effects on the results in our current study. Indeed, further analysis showed that the adjustment of age and sex had no dramatic influence on the significant DMP and gene signatures we identified, such as *FLNC* cg20186636. However, the standard correction procedure might not completely eliminate the effects of age and sex. Thus, the identified DMP signatures and related genes need to be further verified in expanded subject populations in our future studies. Third, our results indicated that the characteristics of blood leukocyte DNA methylation was significantly changed within a subset of genes, which may be enriched in the cerebral cortex (Figures 8E, F). Although communications existed between the brain tissue and peripheral blood, particularly in the status of disease; however, not all alterations of MCI- and AD-associated DNA methylation found in the blood cells may occurred in the brain tissue and functionally participated in AD pathology. Hence, *postmortem* human brain tissue biospecimens need to be investigated to dissect the underlying signature genes involved in AD-related processes that occur in the brain. Fourth, our study only investigated the cross-sectional cohorts of MCI and AD, it is essential to conduct long-term clinical follow-up study to observe the conversion of MCI to AD, as well as identify the preclinical changes of MCI and AD subjects. This could lead us to reveal the *bona fide* DNA methylation-based signatures associated with the course of AD, which might serve as a biomarker for early diagnosis and therapeutic targets of AD. Fifth, it is well known that the amount of specific blood cell types is mildly altered in AD and MCI (Lunnon et al., 2012). Even though the proportions of different blood cells have been controlled in this study, single types of blood cell or a single-cell DNA methylome strategy should be more suitable for identifying MCI- and AD-associated signatures (Karemaker and Vermeulen, 2018). Sixth, our present study has identified a number of potential DNA methylation-based biosignatures of MCI and AD, but the exact role of these signature genes in cognitive function impairment and AD etiopathology is not clear. In the future, the AD relevance of these DNA methylation biomarkers should be investigated in parallel with functional studies of novel cognitive decline-associated genes should be carried out *in vitro* and in animal models to provide stronger evidence to support differential DNA methylation modulation of AD pathogenesis. This could enhance a deeper understanding of epigenetic and environmental stimuli of cognitive deterioration and AD pathology.

In summary, our study suggested significant differences in the global methylome profiles in the genomes of blood leukocytes between Chinese patients with MCI/AD and cognitively healthy controls. We report multiple differentially methylated CpG positions related to AD, of which epigenetically dysregulated signature genes such as *RHOJ* were highlighted. Given that the pathophysiology of AD dementia initiates many years, even decades, before overt clinical symptoms (Sperling et al., 2011). Hence, the identification of a reliable biomarker is crucial for timely and effective interventional strategies. The findings of current study might contribute to determine novel potential blood DNA methylation-based biomarkers for the diagnosis of AD onset and progression in Chinese populations.

## Figures and Tables

**Table:** 

**ABCA7**	ATP binding cassette subfamily A member 7
**Aβ**	amyloid β peptide
**AD**	Alzheimer’s disease
**AFAP1**	actin filament associated protein 1
**APOE**	apolipoprotein E
**APP**	amyloid precursor protein
**ANK1**	ankyrin 1
**ANKH**	ANKH inorganic pyrophosphate transport regulator
**B3GALT4**	beta-1,3-galactosyltransferase 4
**BACE1**	β-secretase 1
**BH**	Benjamini–Hochberg procedure
**BIN1**	amphiphysin II
**CASS4**	Cas scaffold protein family member 4
**CD2AP**	CD2 associated protein
**CD33**	CD33 molecule
**CDH23**	cadherin related 23
**CELF1**	CUGBP Elav-like family member 1
**CpG**	cytosine-phosphate-guanine site
**CLU**	clusterin
**CR1**	complement C3b/C4b receptor 1
**CRY1**	cryptochrome circadian regulator 1
**CTSB**	cathepsin B
**CTSD**	cathepsin D
**DDT**	D-dopachrome tautomerase
**DMP**	differentially methylated position
**DMR**	differentially methylated region
**DSG2**	desmoglein 2
**EPHA1**	EPH receptor A1
**EWAS**	epigenome-wide association study
**FERMT2**	FERM domain containing kindlin 2
**FLNC**	filamin C
**GO**	gene ontology
**GWAS**	genome-wide association study
**HLA-DRB1**	major histocompatibility complex, class II, DR beta 1
**HLA-DRB5**	major histocompatibility complex, class II, DR beta 5
**HOXB6**	homeobox B6
**INPP5D**	inositol polyphosphate-5-phosphatase D
**KCNT1**	potassium sodium-activated channel subfamily T member 1
**KEGG**	Kyoto Encyclopedia of Genes and Genomes
**LMF2**	lipase maturation factor 2
**MCI**	mild cognitive impairment
**MEF2C**	myocyte enhancer factor 2C
**MS4A6A**	membrane spanning 4-domains A6A
**MS4A4E**	membrane spanning 4-domains A4E
**N_Shelf**	the region of 2.0–4.0 kilo base-pairs sequence upstream CpG island
**N_Shore**	the region of 2.0 kilo base-pairs sequence upstream CpG island
**NCAPH2**	non-SMC condensing II complex subunit H2
**NEP**	neprilysin
**NME8**	NME/NM23 family member 8
**NRD1**	nardilysin convertase
**OpenSea**	the sequence region located >4.0 kilo base pairs from a CpG island
**PARK2**	parkin RBR E3 ubiquitin protein ligase
**PCA**	principal component analysis
**PER1**	period circadian regulator 1
**PICALM**	phosphatidylinositol binding clathrin assembly protein
**PLS-DA**	partial least squares-discriminant analysis
**PPI**	protein-protein interaction
**PSEN1**	presenilin 1 gene
**PSEN2**	presenilin 2 gene
**PTK2B**	protein tyrosine kinase 2 beta
**RHBDF2**	rhomboid 5 homolog 2
**RHOA**	Ras homolog gene family member A
**RHOJ**	Ras homolog gene family member J
**RIN3**	Ras and Rab interactor 3
**ROC**	receiver operating characteristic
**RPL13**	ribosomal protein L13
**S_Shelf**	the region of 2.0–4.0 kilo base-pairs sequence downstream CpG island
**S_Shore**	the region of 2.0 kilo base-pairs sequence downstream CpG island
**SLC24A4**	solute carrier family 24 member 4
**SORL1**	sortilin-related receptor, L (DLR class) 1
**SP4**	Sp4 transcription factor
**SYN3**	synapsin III
**SYT7**	synaptotagmin 7
**TSS200**	the upstream 200 base-pairs sequence of the transcription start site
**TSS1500**	the upstream 1500 base-pairs sequence of the transcription start site
**UQCRC1**	ubiquinol-cytochrome c reductase core protein 1
**ZADH2**	also known as PTGR3, prostaglandin reductase 3
**ZCWPW1**	zinc finger CW-type and PWWP domain containing 1

## Data Availability

The datasets presented in this study can be found in online repositories. The names of the repository/repositories and accession number(s) can be found in the article/Supplementary Material. The raw DNA methylome data used in this study is publicly available on the Gene Expression Omnibus database with the accession number GSE208623. Further inquiries can be directed to the corresponding authors.

## Supplementary material

SUPPLEMENTARY FIGURE S1 An overview of the methodological flow.

SUPPLEMENTARY FIGURE S2 Singular value decomposition analysis of the effects of confounders. The effects of all confounders, including age, sex, sample group, sample well, slide, and array, were investigated by performing singular value decomposition (SVD) analysis via champ. SVD() function.

SUPPLEMENTARY FIGURE S3 PCA and PLS-DA models for separating Alzheimer’s disease, mild cognitive impairment, and cognitively healthy controls. **(A)** PCA plot. For X variable dataset, model interpretability R2X = 0.223. **(B)** PLS-DA plot. The respective model interpretability for X and Y variable dataset was R2X = 0.340 and R2Y = 0.613, model predictability Q2 = 1.000. AD: Alzheimer’s disease; MCI: mild cognitive impairment; CHCs: cognitively healthy controls.

SUPPLEMENTARY FIGURE S4 Manhattan plot showing the top hits for AD and MCI group. **(A)** AD versus CHCs group, **(B)** MCI versus CHCs group, **(C)** AD versus MCI group. Manhattan plot of all probes across the whole genome illustrating P values (Y-axis, -log10 scale) against genomic location (X-axis). Chromosomes are distinguished by different colors. The red horizontal solid line represents –log10(6.68×10-8) (corresponding to the Bonferroni adjusted P value = 0.05).

SUPPLEMENTARY FIGURE S5 Volcano plot of significant differential methylated positions between AD, MCI and CHCs. **(A)** Differentially methylated positions between AD and CHCs, Padj value < 0.05, |Delta beta| cutoff > 0.2. 272 hypermethylated and 179 hypomethylated positions. **(B)** Differentially methylated positions between MCI and CHCs, Padj value < 0.05, |Delta beta| cutoff > 0.2. 1100 hypermethylated and 1327 hypomethylated positions. **(C)** Differentially methylated positions between AD and MCI, Padj value < 0.05, |Delta beta| cutoff > 0.2. 574 hypermethylated and 439 hypomethylated positions.

SUPPLEMENTARY FIGURE S6 Bar plots demonstrating the functional genomic regions distribution patterns of differentially methylated positions. **(A)** and **(B)** represents the number of various genomic regions harboring significant (Padj value < 0.05) hypermethylated and hypomethylated positions from the comparative group of AD versus CHCs, respectively. **(C)** and **(D)** represents the number of various genomic regions harboring significant (Padj value < 0.05) hypermethylated and hypomethylated positions from the comparative group of MCI versus CHCs, respectively. **(E)** and **(F)** represents the number of various genomic regions harboring significant (Padj value < 0.05) hypermethylated and hypomethylated positions from the comparative group of AD versus MCI, respectively. Colors represent different regions. TSS1500 and TSS200 means the upstream 1500 and 200 base-pairs of the transcription start site (TSS), respectively; 5´UTR means the region at the 5’ end of a mature transcript preceding the initiation codon that is not translated into protein; 3´UTR means the region at the 3’ end of a mature transcript preceding the stop codon that is not translated into protein; 1stExon means the first exon of certain gene; Body means the sequence from the initiation codon to the stop codon.

SUPPLEMENTARY FIGURE S7 Ternary plots demonstrating the genomic distribution pattern of significant CpG sites. **(A)** and **(B)** shows the genomic distribution pattern of significantly (Padj < 0.05) hypermethylated and hypomethylated CpG sites of the three comparative groups of AD versus CHCs, MCI versus CHCs, and AD versus MCI, respectively.

SUPPLEMENTARY FIGURE S8 The common 441 differentially methylated positions overlapped by the three comparative groups. **(A)** Total of 441 differentially methylated positions were shared by the comparative groups of AD versus CHCs, MCI versus CHCs, and AD versus MCI. **(B)** The common 441 DMPs were aligned to 213 unique genes.

SUPPLEMENTARY FIGURE S9 Violin plots of differentially methylated CpG positions compared between AD, MCI, and CHCs group after age and sex adjustment. **(A)** FLNC cg20186636. **(B)** PARK2 cg09656629. **(C-E)** RHOJ cg18771300, cg07189587, and cg07157030. **(F)** cg22721608. **(G)** cg24361198. **(H)** AFAP1 cg06758191. **(I)** ANKH cg02821156, and **(J)** cg15970769. Patients with AD and MCI, and CHCs are labeled with red, purple, and green, respectively. The Y-axis represents the beta value of each CpG site. BH procedure was used for correction to obtain Padj value. *: Padj < 0.05, **: Padj < 0.01, ***: Padj < 0.001.

SUPPLEMENTARY FIGURE S10 ROC analysis of several CpG positions for predicting AD event. Receiver operating characteristic (ROC) curves analysis of 7 DMPs including cg15970769, cg24361198, cg18771300, cg02821156, cg07157030, cg09656629, and cg07189587 in predicting the transition of MCI to AD. ROC analysis was performed using pROC in the R package.

SUPPLEMENTARY FIGURE S11 Protein-protein interaction analysis of all proteins encoded by the common 213 genes. Protein-protein interaction (PPI) analysis was performed on all proteins encoded by the overlapping 213 genes between AD versus CHCs, MCI versus CHCs, and AD versus MCI. PPI analysis was carried out by using online tool STRING.

SUPPLEMENTARY FIGURE S12 Transcription factor binding motif enrichment analysis of the overlapping 441 DMPs. Transcription factor motif enrichment analysis of the 441 differentially methylated positions (+-100 bp) in 213 genes. +-100bp sequences around target CpG were acquired by using hg19(GRch37).
