# Supplementary figures and images for: Altered DNA methylome profiles of blood leukocytes in Chinese patients with mild cognitive impairment and Alzheimer’s disease
Source: Front Genet. 2023 Jun 14;14:1175864. doi: 10.3389/fgene.2023.1175864 (PMC10300350; doi:10.3389/fgene.2023.1175864)

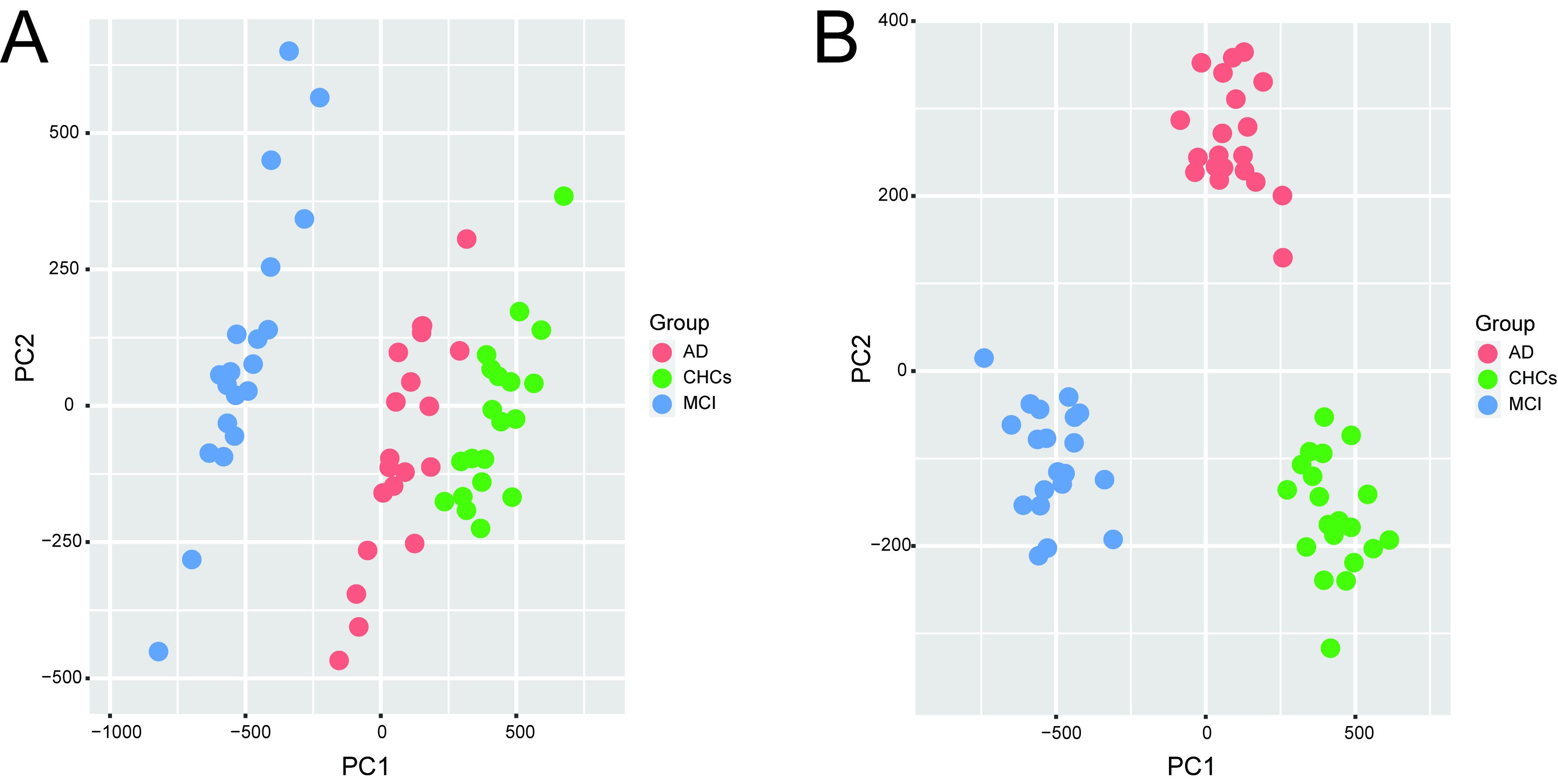

Supplement: Supplementary file 5 [file Image3.TIF]

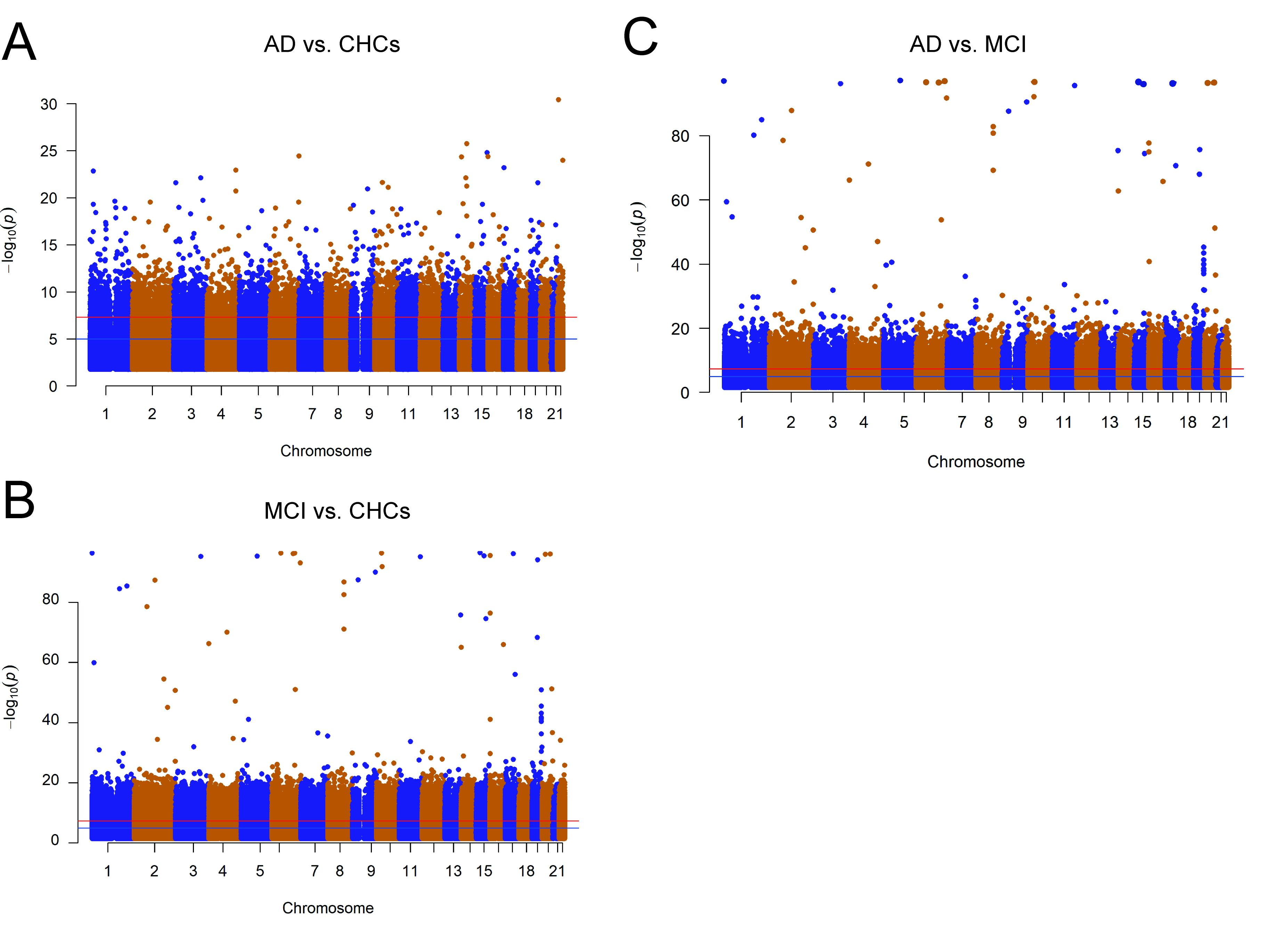

Supplement: Supplementary file 6 [file Image4.TIF]

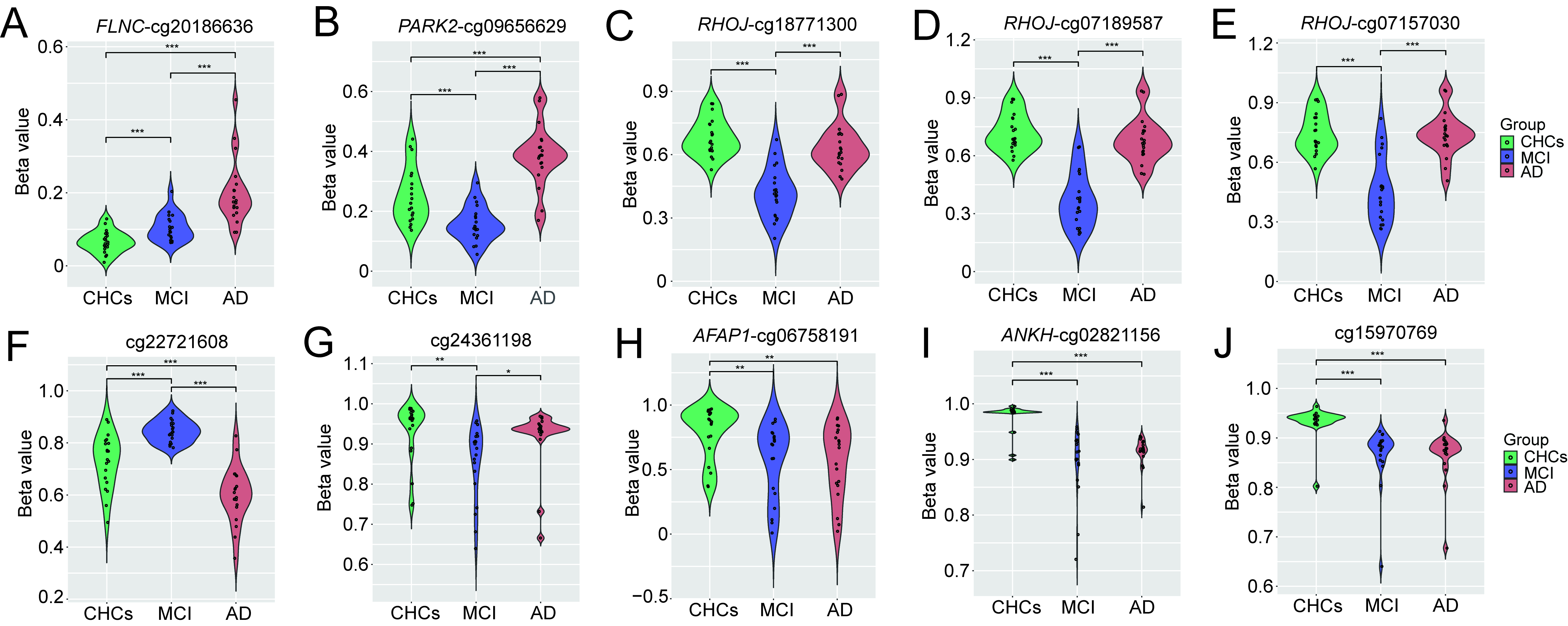

Supplement: Supplementary file 7 [file Image9.TIF]

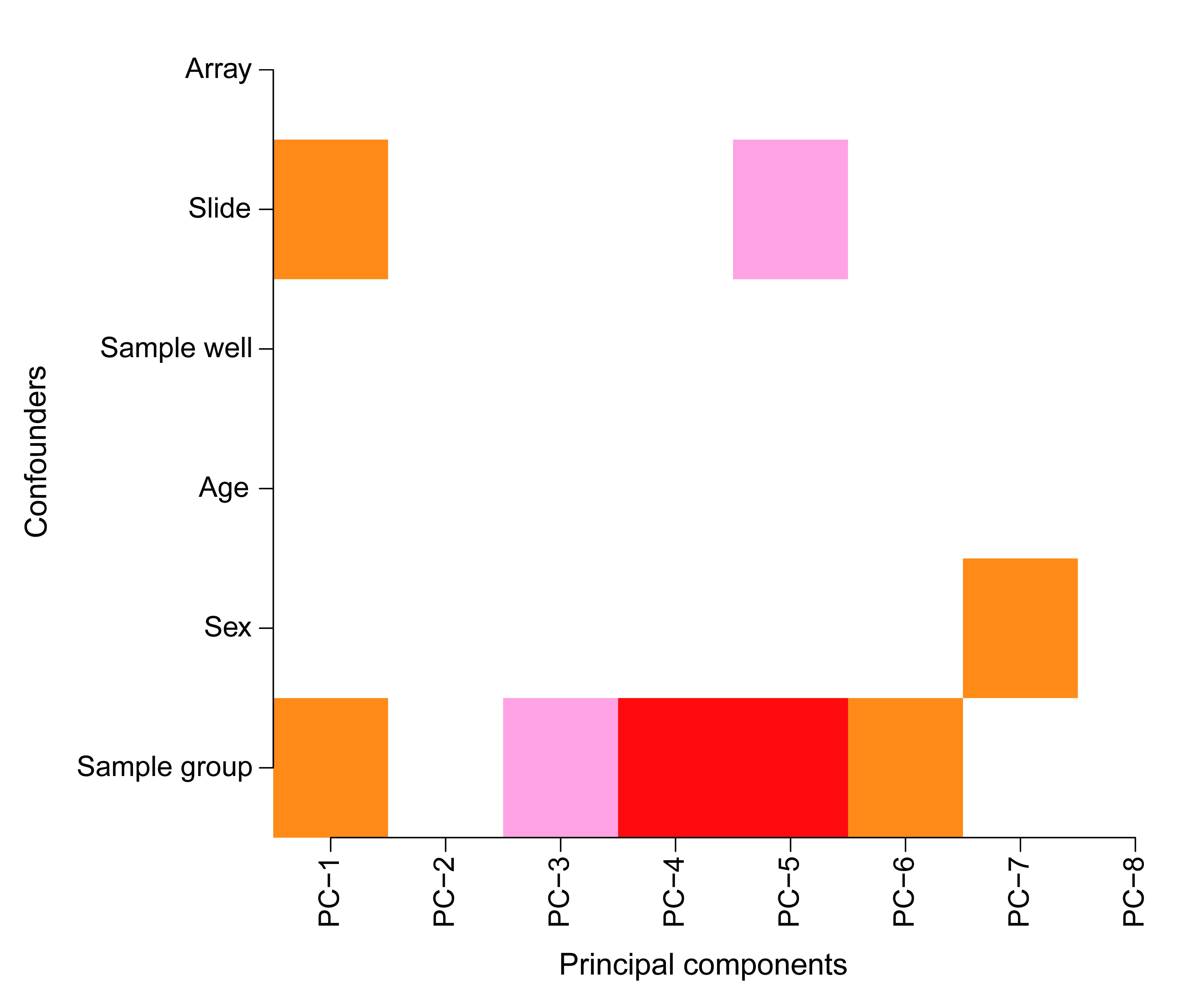

Supplement: Supplementary file 8 [file Image2.TIF]

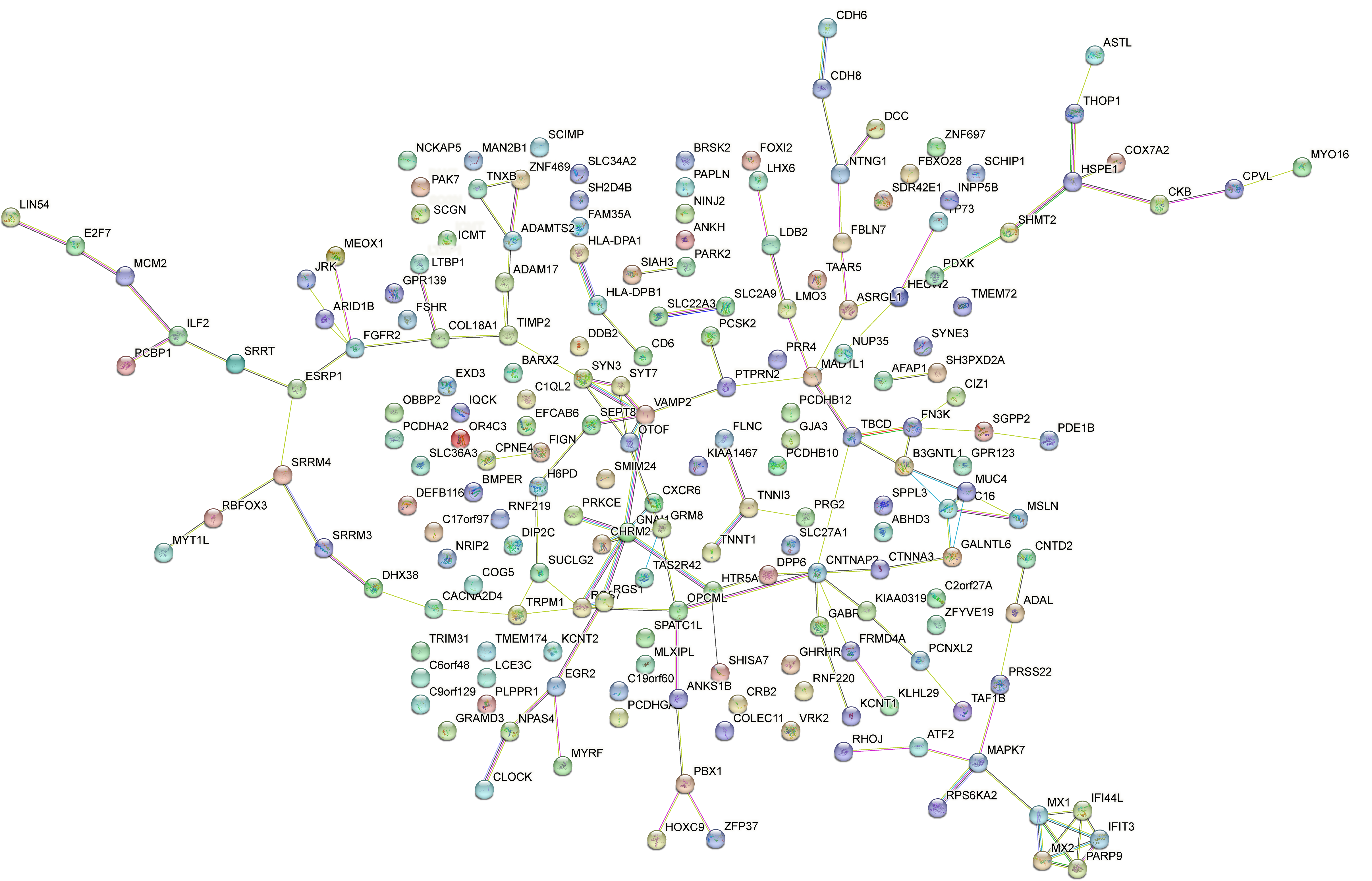

Supplement: Supplementary file 9 [file Image11.TIF]

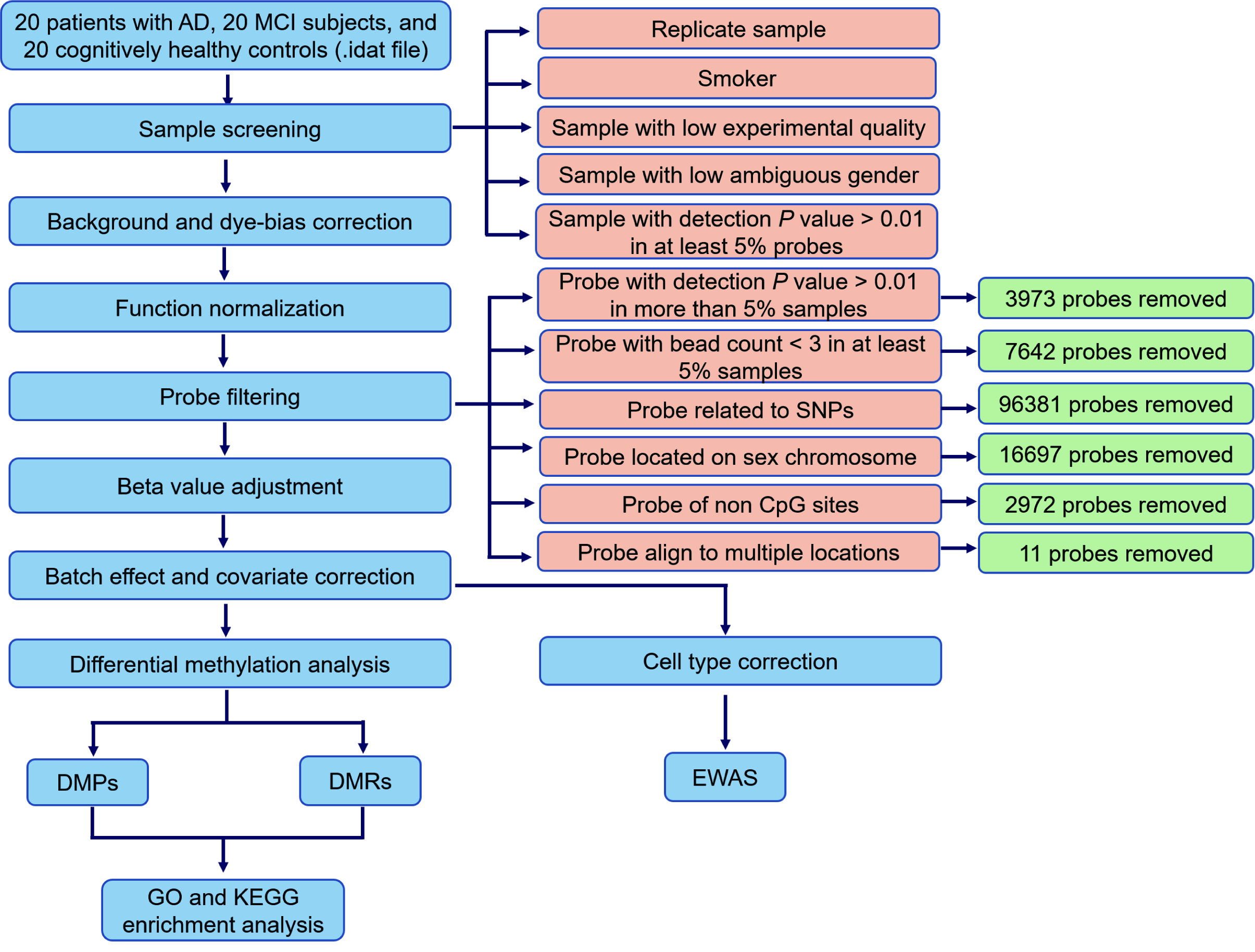

Supplement: Supplementary file 10 [file Image1.TIF]

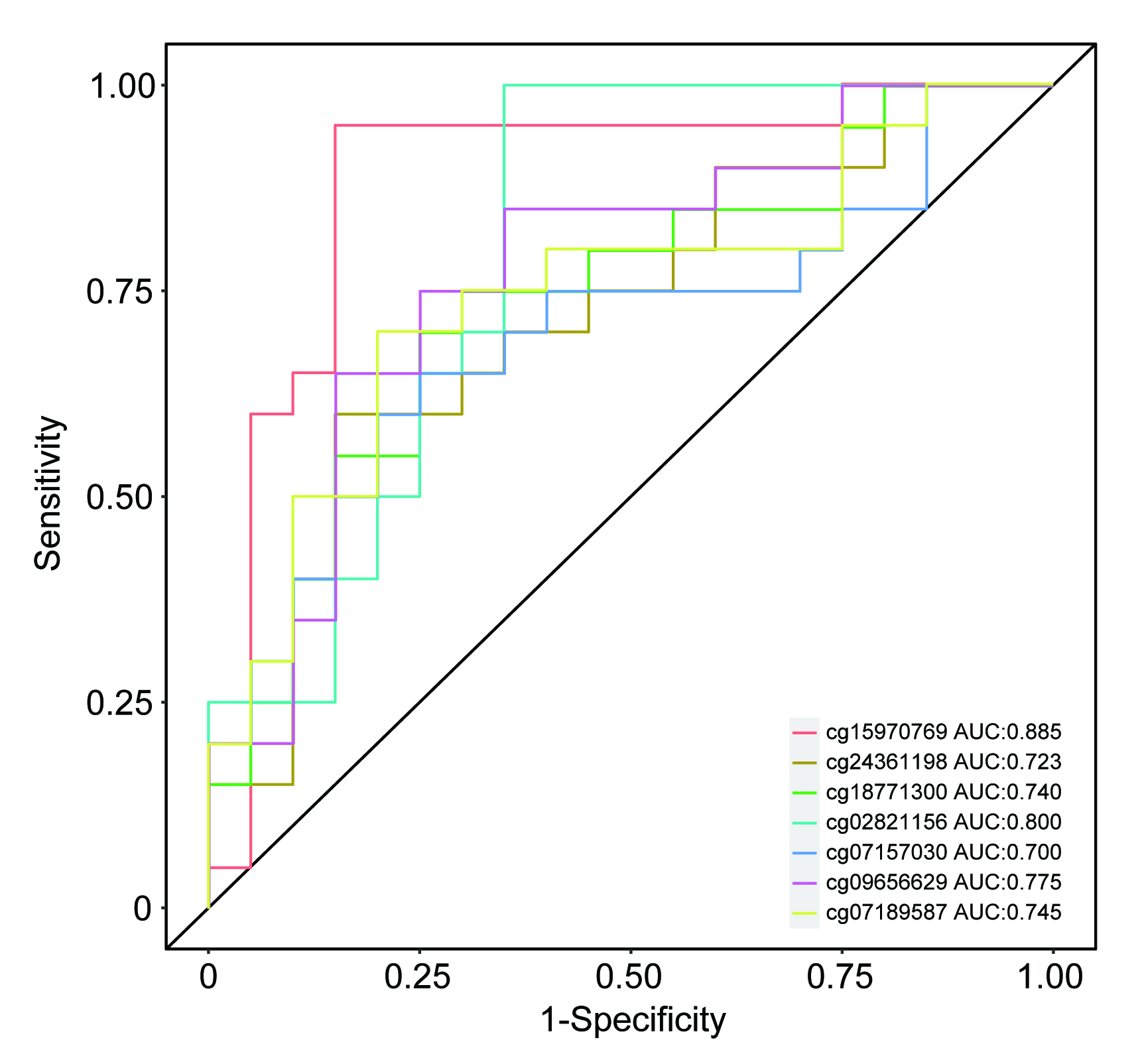

Supplement: Supplementary file 11 [file Image10.TIF]

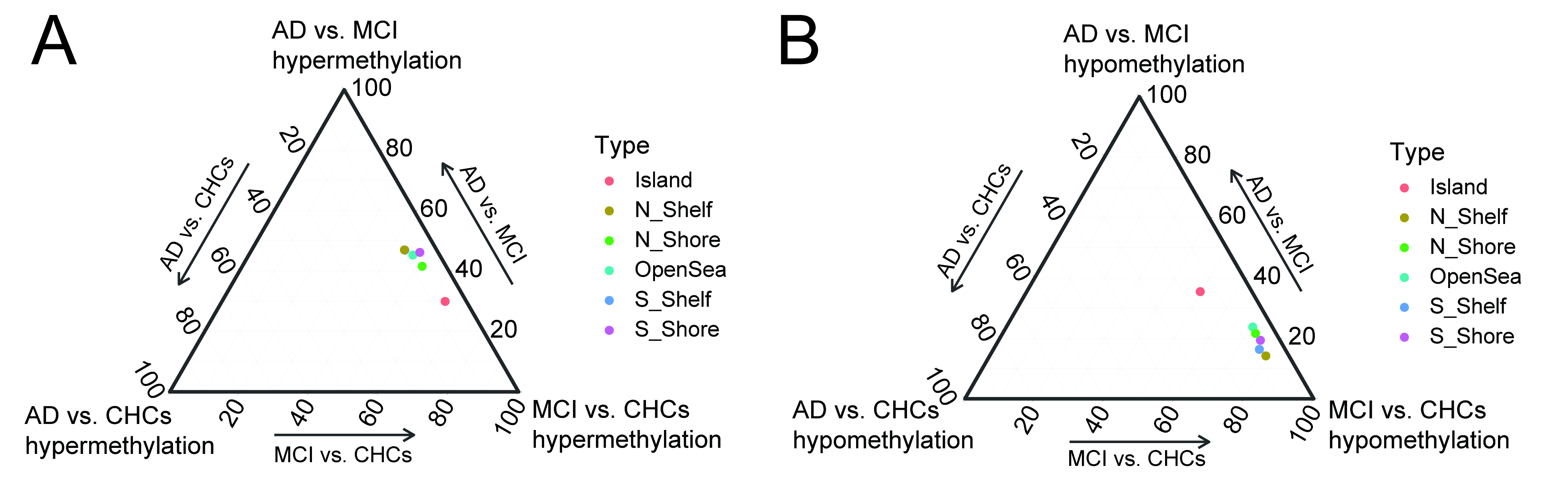

Supplement: Supplementary file 12 [file Image7.TIF]

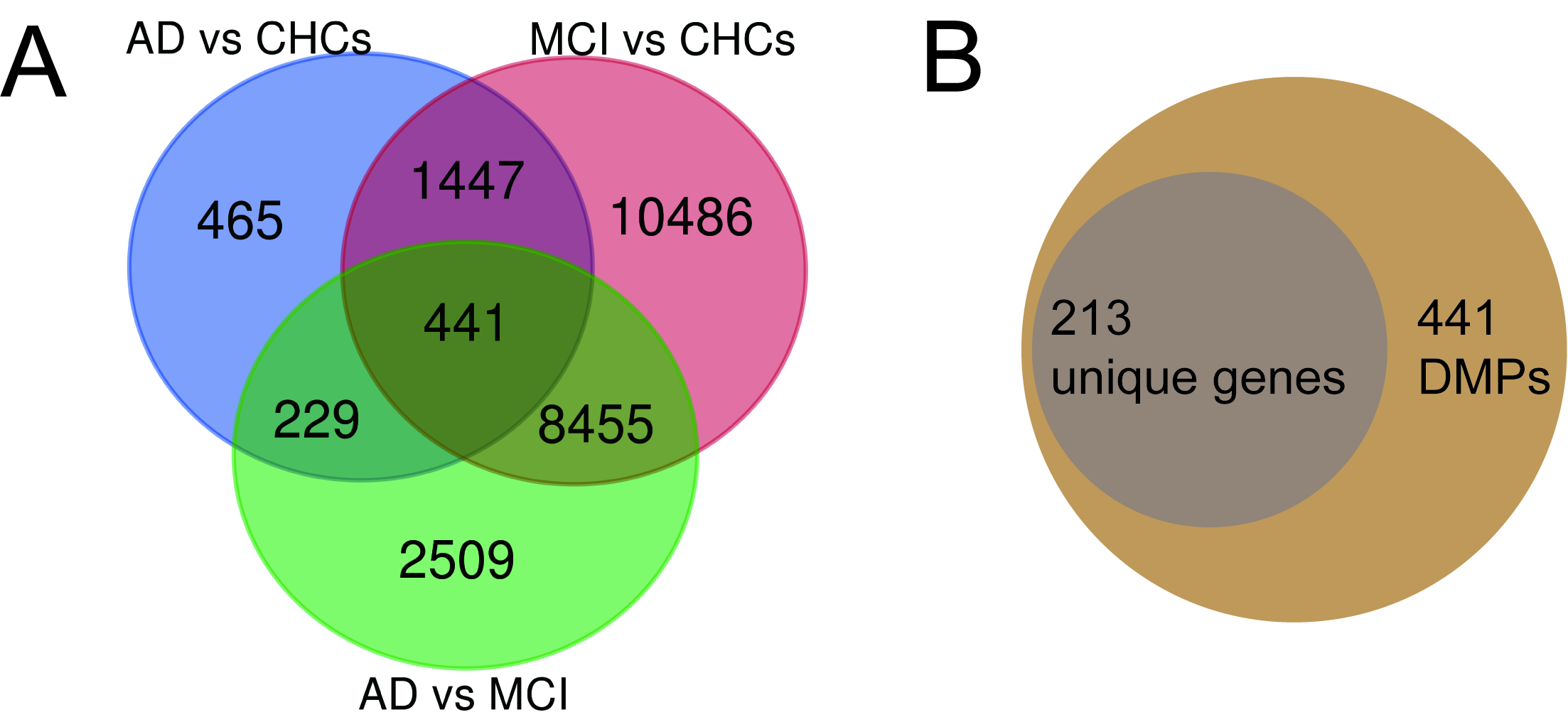

Supplement: Supplementary file 17 [file Image8.TIF]

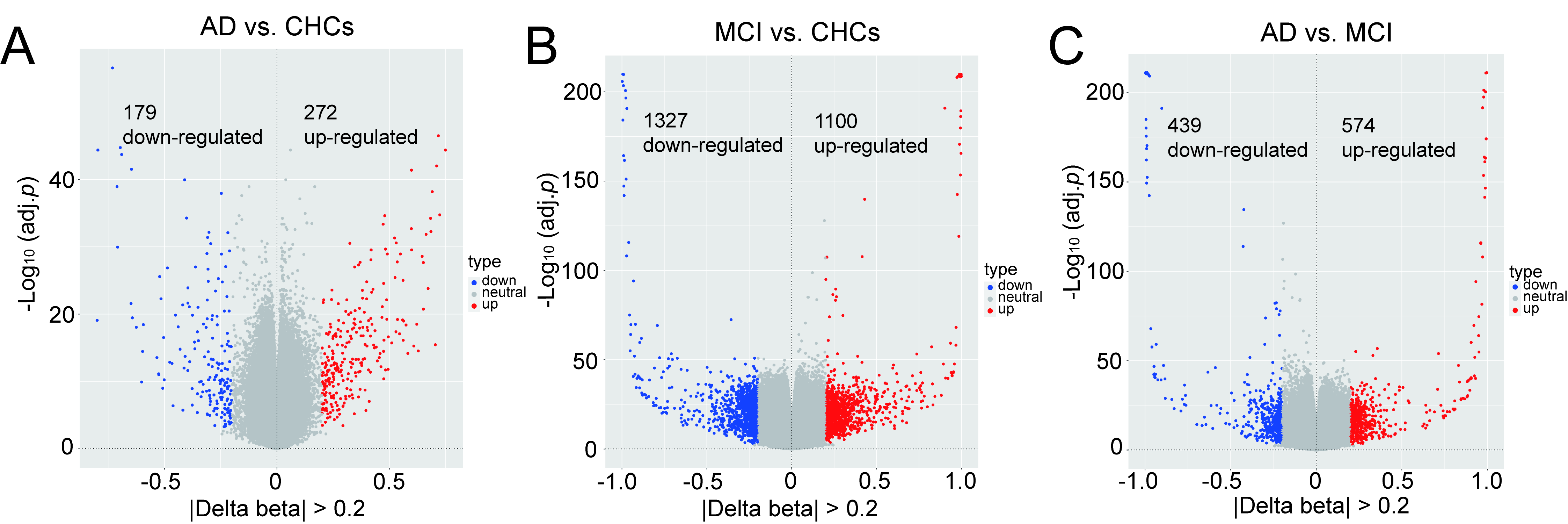

Supplement: Supplementary file 20 [file Image5.TIF]

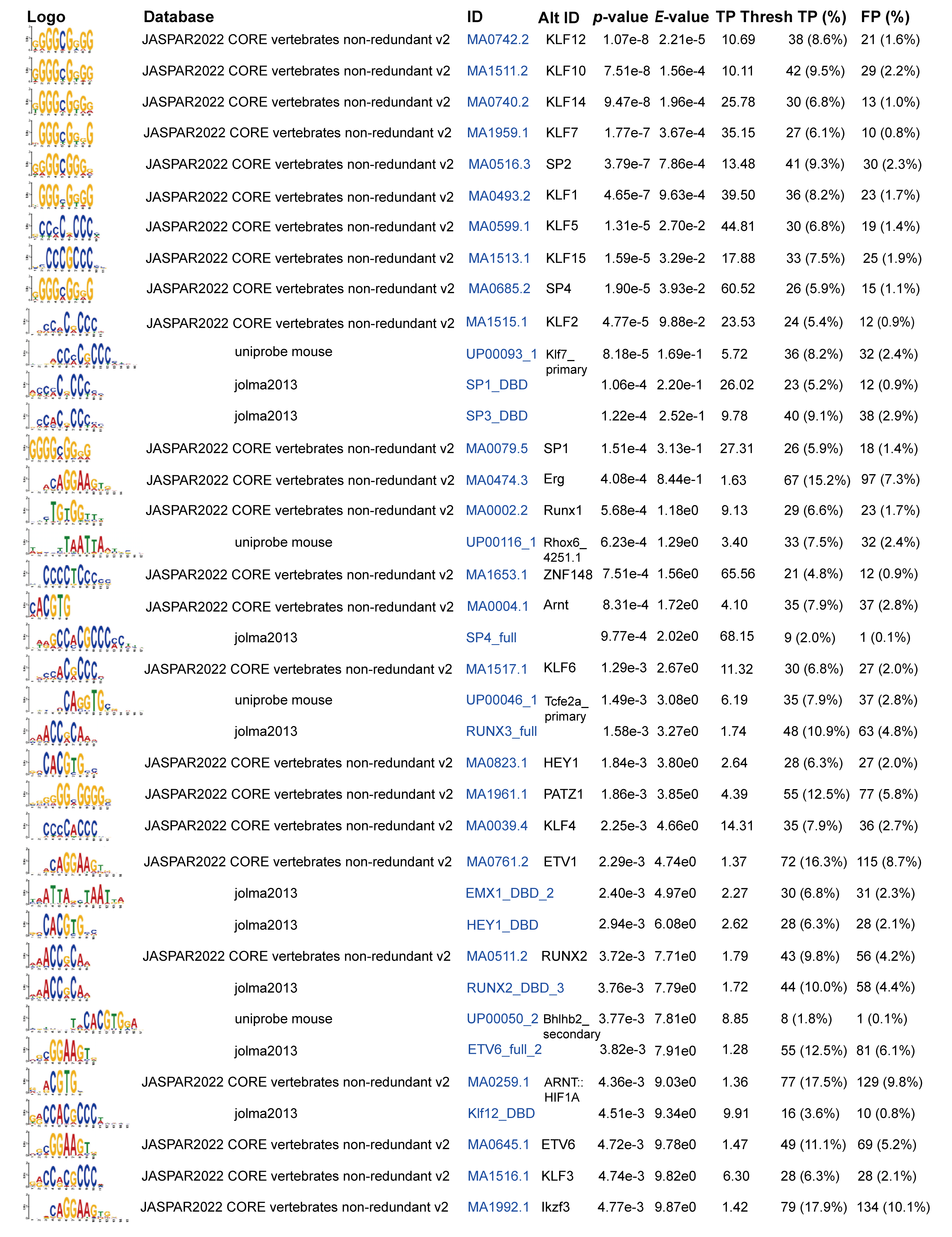

Supplement: Supplementary file 22 [file Image12.TIF]
